# The high‐quality genome of diploid strawberry (*Fragaria nilgerrensis*) provides new insights into anthocyanin accumulation

**DOI:** 10.1111/pbi.13351

**Published:** 2020-02-15

**Authors:** Junxiang Zhang, Yingying Lei, Baotian Wang, Song Li, Shuang Yu, Yan Wang, He Li, Yuexue Liu, Yue Ma, Hongyan Dai, Jiahong Wang, Zhihong Zhang

**Affiliations:** ^1^ Liaoning Key Laboratory of Strawberry Breeding and Cultivation College of Horticulture Shenyang Agricultural University Shenyang China; ^2^ Biomarker Technologies Corporation Beijing China

**Keywords:** *Fragaria nilgerrensis*, PacBio SMRT, Hi‐C, transposable element, *MYB10*, promoter activity

## Abstract

*Fragaria nilgerrensis* is a wild diploid strawberry species endemic to east and southeast region in Asia and provides a rich source of genetic variations for strawberry improvement. Here, we present a chromosome‐scale assembly of *F. nilgerrensis* using single‐molecule real‐time (SMRT) Pacific Biosciences sequencing and chromosome conformation capture (Hi‐C) genome scaffolding. The genome assembly size was 270.3 Mb, with a contig N50 of ∼8.5 Mb. A total of 28 780 genes and 117.2 Mb of transposable elements were annotated for this genome. Next, detailed comparative genomics with the high‐quality *F. vesca* reference genome was conducted to obtain the difference among transposable elements, SNPs, Indels, and so on. The genome size of *F. nilgerrensis* was enhanced by around 50 Mb relatively to *F. vesca*, which is mainly due to expansion of transposable elements. In comparison with the *F*. *vesca* genome, we identified 4 561 825 SNPs, 846 301 Indels, 4243 inversions, 35 498 translocations and 10 099 relocations. We also found a marked expansion of genes involved in phenylpropanoid biosynthesis, starch and sucrose metabolism, cyanoamino acid metabolism, plant–pathogen interaction, brassinosteroid biosynthesis and plant hormone signal transduction in *F. nilgerrensis*, which may account for its specific phenotypes and considerable environmental adaptability. Interestingly, we found sequence variations in the upstream regulatory region of *FnMYB10*, a core transcriptional activator of anthocyanin biosynthesis, resulted in the low expression level of the *FnMYB10* gene, which is likely responsible for white fruit phenotype of *F. nilgerrensis.* The high‐quality *F. nilgerrensis* genome will be a valuable resource for biological research and comparative genomics research.

## Introduction

Strawberries are one of the most economically important berry fruits throughout the world. The main cultivated strawberry species, *Fragaria* × *ananassa*, is derived from a hybrid of two wild octoploid species, *F. chiloensis* and *F. virginiana* (Bolger *et al.*, 2014; Darrow, 1966). Strawberry belongs to Rosaceae family of the genus *Fragaria* which appears to be closely related to *Potentilla* and *Duchesnea* (Harrison and Luby, 1997). Recent studies show that cultivated strawberry is formed through the incorporation of four diploid progenitor species and the four ancestors are *F. iinumae*, *F. nipponica*, *F. viridis* and *F. vesca* (Edger *et al.*, 2019).

A total of 24 wild species of *Fragaria* have been identified (Folta and Davis, 2006; Staudt, 2009), including 12 diploids (2*n* = 2*x* = 14), 5 tetraploids (2*n* = 4*x* = 28), 1 pentaploid (2*n* = 5*x* = 35), 1 hexaploid (2*n* = 6*x* = 42), 3 octoploids (2*n* = 8*x* = 56) and 2 decaploid (2*n* = 10*x* = 70). The formation of higher‐polyploid wild species results from the interfertility between and within ploidy levels (Potter *et al.*, 2000). These wild species are mainly distributed in Asia, Europe and America (Staudt, 1989, 1999). A number of valuable characteristics, such as cold tolerance, disease resistance, firm fruit, high aroma, different fruit colours and so on, exist in wild species of *Fragaria* that can be used for the cultivated strawberry (Darrow, 1966; Guo *et al.*, 2017; Hancock, 1999; Luo *et al.*, 2018). Strawberry breeders are focused on improving stress tolerance, fruit quality, productivity, disease resistance and developing new strawberry cultivars. Some desirable traits from lower ploidy species have been introduced into cultivated strawberry through pollinations via unreduced gametes or doubling the chromosome by colchicine treatment (Bors and Sullivan, 1998; Noguchi et al. 2002; Sangiacomo and Sullivan, 1994).

China has been considered as the crucial geographical distribution centre of wild strawberry resources in the world. Among 24 recognized *Fragaria* species, 13 wild species of *Fragaria* are distributed in China (Guo *et al.*, 2017; Lei *et al.*, 2017; Sargent *et al.*, 2004; Staudt, 2009; Wang *et al.*, 2017). Here, we assembled the diploid strawberry, *F. nilgerrensis*, which is from Yunnan, China. *F. nilgerrensis* is a self‐compatible and sympodial‐runnering (Staudt, 1989). F_1_ plants from *F. nilgerrensis* and *F. vesca* are all stunted and failed to flowering (Sargent *et al.*, 2004). Multiple literatures also suggest that *F. nilgerrensis* is highly divergent from *F. vesca* (Liu *et al.*, 2016; Njuguna *et al.*, 2013; Potter *et al.*, 2000; Qiao *et al.*, 2016; Rousseau‐Gueutin et al. 2009).

The genotype and phenotype of *F. nilgerrensis* are quite different from *F. vesca*. The karyotype of *F. nilgerrensis* is composed of two pairs of 45S rDNA and one pair of 5S rDNA according to fluorescence in situ hybridization method, whereas *F. vesca* generally consists of 3 pairs of 45S and 1 pair of 5S (Nathewet *et al.*, 2009; Rho *et al.*, 2012). The karyotype formula of *F. nilgerrensis* is 8m + 6sm, while the karyotype formula of *F. vesca* is 10m + 4sm (Nathewet *et al.*, 2009). *F. nilgerrensis* is robust and has heavy pubescence on all plant compared with *F. vesca* (Yarnell, 1928). The mature fruits of *F. nilgerrensis* typically have banana‐like and melon‐ or peach‐like aromas (Noguchi et al. 2002) and the scapes of *F. nilgerrensis* are short with respect to *F. vesca* (Yarnell, 1928). The leaves of *F. nilgerrensis* are leathery and thick, and the leaves of *F. vesca* often curl at edges. The petioles of *F. nilgerrensis* are thick and leathery, while the petioles of *F. vesca* are gracile. The calyx of *F. nilgerrensis* is clasping and the calyx of *F. vesca* is reflexed or spreading (Lei *et al.*, 2017). The berries of *F. nilgerrensis* are subglobose and achenes are very small and extremely sunken, whereas the berries of *F. vesca* are long conic and achenes are red and raised (Lei *et al.*, 2017). In addition, *F. nilgerrensis* has many important traits, such as waterlogging tolerance and leaf disease resistance (Guo *et al.*, 2017). Despite *F. nilgerrensis* has many differences in many important traits compared with *F. vesca*, the reasons for the phenotype differences, resistance and utilization of *F. nilgerrensis* for genetic improvement of cultivated strawberry are still largely lacking. Thus, a finished and accurate reference genome of *F. nilgerrensis* will lay a solid foundation for understanding the genomic evolution of *Fragaria* and identifying functional genes for important traits. Here, we present a high‐quality reference genome of the *F. nilgerrensis* using a combination of sequencing technologies. We used this genome to conduct a comparative genomic analysis with the reported *F. vesca* genome. We also identified the key factor that is associated with the white fruit phenotype of *F. nilgerrensis*. This *F. nilgerrensis* genome assembly will provide a valuable resource for further molecular functional analyses of this species and comparative genomics research across Rosaceae family.

## Results

### Genome sequencing and assembly

*Fragaria* *nilgerrensis* used for sequencing is a self‐compatible, white fruit and sympodial‐runnering (Figure S1). A combination of Illumina short‐read sequencing from Illumina HiSeq, PacBio long‐read sequencing technology and high‐throughput chromosome conformation capture (Hi‐C) sequencing was used to assemble the *F. nilgerrensis* genome (Figure S2). Our sequencing of *F. nilgerrensis* resulted in coverage of ~ 76.77‐fold PacBio single‐molecule long reads (22.65 Gb with an average length of 10.96 kb), 66.48‐fold Illumina paired‐end short reads (19.62 Gb) and 55.88‐fold Hi‐C data (15.09 Gb). The genome assembly was conducted in a stepwise fashion (Zhang *et al.*, 2019). Falcon and Canu (Koren *et al.*, 2017) pipelines were used for the initial assembly of the PacBio sequencing data, yielding 389 contigs (N50 of ~8.50 Mb). To anchor the scaffolds to chromosomes, we performed Hi‐C libraries of *F. nilgerrensis* (Figure S3), generating 15.09 Gb Hi‐C pair‐end reads. HiC‐Pro software (Servant *et al.*, 2015) was used for duplicate removal, sorting and quality assessment, and LACHESIS software (Hariharan and Toyama, 2004) was applied to obtain uniquely mapped valid reads for Hi‐C scaffolding. As a result, 264.6 Mb (97.89%) of the assembly was placed on seven chromosome groups. Next, gap filling for the Hi‐C assembled sequence data was conducted by the PBjelly pipeline (English *et al.*, 2012). The final assembly contained 257 scaffolds and 430 contigs, with scaffold N50 value of 38.3 Mb and a contig N50 of 8.5 Mb, respectively. The total assembly size is 270.3 Mb with maximum scaffold and contig lengths of 50.64 and 20.71 Mb, respectively. Based on a k‐mer analysis (k = 19), we evaluated the genome with a heterozygosity of 0.1% and GC content of 39.22% (Figure S4).

### Assessment of genome quality

To test the accuracy and completeness of the assembly, we combined three different methods: short‐read sequencing alignment, computational pipelines Benchmarking Universal Single‐Copy Orthologs (BUSCO) (Simão *et al.*, 2015) and Core Eukaryotic Genes Mapping Approach (CEGMA) (Parra *et al.*, 2007) assessment. BWA‐MEM pipeline was used for aligning Illumina paired‐end (PE) reads to our assembly and 97.08% Illumina paired‐end reads could properly mapped to PacBio‐based assembly genome with the correct orientation and estimated insert size (Table S1). We also used the BUSCO method based on a benchmark of 1440 conserved plant genes to assess the completeness of gene regions, of which 95% had complete gene coverage (including 5.28% duplicated ones), 2.22% were fragmented and 8.06 % were missing (Table S2). Moreover, the completeness of the assembly was investigated by searching for 458 core eukaryotic genes (CEGs). In total, 450 of 458 CEGs were completely present, indicating that fewer than 2% of the CEGs could not be detected, which confirms the high completeness of the assembly (Table S3). Finally, the whole‐genome alignment of *F*. *nilgerrensis* and *F*. *vesca* showed strong collinearity and consistency (Figure S5). Therefore, these results show that our genome assembly is of high quality and has high coverage, which provides an opportunity to comprehensive assess genome variations between *F*. *nilgerrensis* and *F*. *vesca*.

### Genome annotation

We annotated the genome using the EVidenceModeler pipeline (Haas *et al.*, 2008) incorporating *ab initio* predictions, homology‐based search and RNA‐Seq data (Figure S6–S7; Table S4), resulting in 28 780 protein‐coding genes in the *F*. *nilgerrensis* genome (Table 1). The numbers of annotated genes in *F*. *nilgerrensis* are close to genes in the *F. vesca* genome which has 28 588 genes (Table 1). In addition, we identified 1567 transcription factors in the *F*. *nilgerrensis* genome. Then, the predicted protein‐coding genes were annotated through blast with the nonredundant (NR), translation of EMBL nucleotide sequence database (TrEMBL), eukaryotic orthologous groups (KOG), Kyoto Encyclopedia of genes and genomes (KEGG) and gene ontology (GO) database and 96.01% of predicted protein‐coding genes could be annotated in these database (Table S5). In the GO analysis, 9890 (34.36 %), 9534 (33.13%) and 6395 (22.22 %) annotated protein‐coding genes were assigned to the GO slim terms biological process, molecular function and cellular component, respectively (Figure S8).

**Table 1:** The comparison of genome assembly between *Fragaria nilgerrensis and F*. *vesca* (Hawaii‐4)

Assembly feature	*F*. *nilgerrensis*	*F*. *vesca* (Hawaii‐4)
Total assembly size	270.3 Mb	220.4 Mb
Assembly % of genome	97.91	99.8
Repeats region % of assembly	43.40	34.85
Predicted gene models	28 780	28 588
Number of contigs	430	61
Contig N50	8.51 Mb	7.9 Mb
Number of scaffolds	257	31
Scaffold N50	38.3 Mb	36.1 Mb
Chr01 length/genes	23.35 Mb/2443	24.25 Mb/3056
Chr02 length/genes	34.87 Mb/4019	29.35 Mb/4145
Chr03 length/genes	46.88 Mb/4527	38.32 Mb/4612
Chr04 length/genes	38.31 Mb/3820	33.91 Mb/3740
Chr05 length/genes	33.76 Mb/3793	29.43 Mb/3978
Chr06 length/genes	50.64 Mb/5505	39.8 Mb/5449
Chr07 length/genes	28.35 Mb/3384	24.23 Mb/3431
Unanchored	14.14 Mb/1289	1.07 Mb/177

The set of predicted noncoding genes included 302 ribosomal RNAs (rRNAs) belonging to four families, 495 transfer RNAs (rRNA) belonging to 22 families, 58 microRNAs (miRNAs) belonging to 20 families, 81 small nuclear RNA (snRNA) belonging to eight families and 369 small nucleolar RNA (snoRNA) belonging to two families (Table S6). We also identified 2451 pseudogenes in the *F. nilgerrensis* genome using the GeneWise software 7 (Birney and Durbin, 2000).

### Evolution and gene family expansion/contraction analysis

To investigate the evolution of *F. nilgerrensis*, we compared the *F. nilgerrensis* to 13 other plant species. These species include 8 plants in the same Rosaceae order (*F*. *vesca*, *Malus x domestica*, *Prunus avium*, *Prunus persica*, *Pyrus communis*, *Pyrus bretschneideri*, *Rosa chinensis* and *Rubus occidentalis*), four plants in the same eudicots clade (*Arabidopsis thaliana*, *Vitis vinifera*, *Solanum lycopersicum* and *Citrus sinensis*), and *Oryza sativa* as the outgroup.

**Figure 1:**
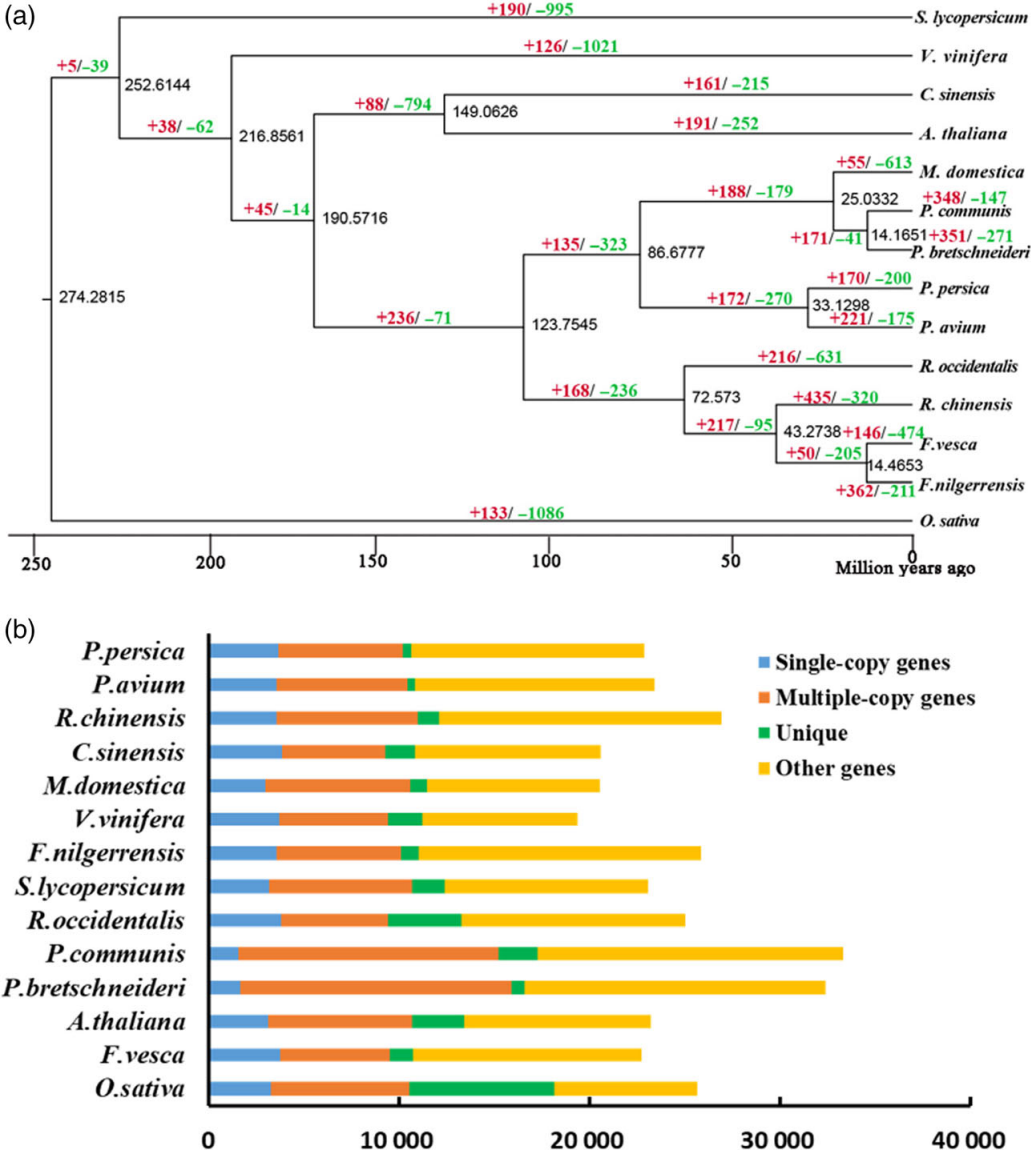
Gene family and genome evolution of *Fragaria nilgerrensis*. (a) The estimation of divergence time and expansion (red), contraction (green) of gene families in *F. vesca*, *Malus × domestica*, *Prunus avium*, *Prunus persica*, *Pyrus Communis*, *Pyrus bretschneideri*, *Rosa chinensis*, *Rubus occidentalis*, *Arabidopsis thaliana*, *Vitis vinifera*, *Solanum lycopersicum*, *Citrus sinensis* and *Oryza sativa*. A phylogenetic tree was performed based on 373 single‐copy orthologous genes using *O*. *sativa* as the outgroup. The numerical value beside each node is the estimated divergent time. (b) The distribution of single‐copy, multiple‐copy, unique and other orthologues in *F*. *vesca*, *M*. *× domestica*, *P*. *avium*, *P*. *persica*, *P*. *communis*, *P*. *bretschneideri*, *R*. *chinensis*, *R*. *occidentalis, A. thaliana*, *V. vinifera*, *S. lycopersicum*, *C. sinensis* and *O*. *sativa.*

A high‐confidence phylogenetic tree of the 14 species was performed using genes extracted from 373 orthologs from single‐copy gene families (Figure 1a; Appendix S1). As expected, *F. nilgerrensis* and its close relatives in Rosaceae (*F*. *vesca*, *M*. *domestica*, *P*. *avium*, *P*. *persica*, *P*. *communis*, *P*. *bretschneideri*, *R*. *chinensis* and *R*. *occidentalis)* were clustered into one monophyletic group and *F. nilgerrensis* had closest relationship with *F. vesca*, *R*. *chinensis* and *R*. *occidentalis* (Figure 1a). This phylogenetic tree is mostly in broad consensus with the species relationships observed in previous report (Saint‐Oyant *et al.*, 2018). It was estimated that *F*. *nilgerrensis* and *F*. *vesca* diverged from approximately 14.46 million years ago (Mya) (Figure 1a). In addition, the *F*. *vesca* and *R*. *chinensis* had diverged from around 43.27 Mya (Figure 1a), which is close to results as described previously (Saint‐Oyant *et al.*, 2018). The proportion of single‐ and multiple‐copy genes of *F*. *nilgerrensis* was similar to the other species genomes apart from *P*. *bretschneideri* and *P*. *communis*, which had a lower proportion of single‐ and multiple‐copy genes (Figure 1b).

**Figure 2:**
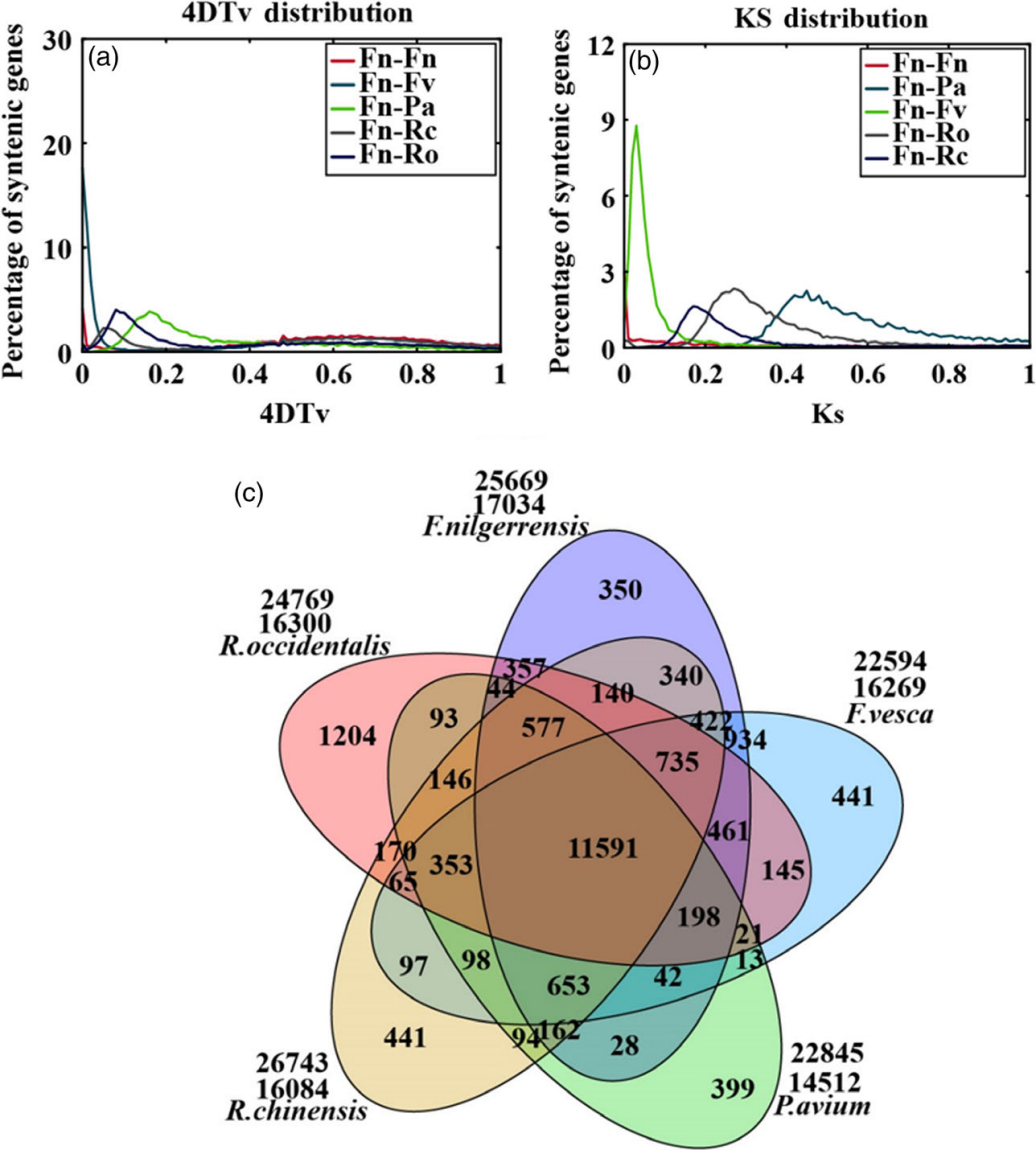
Comparative analysis and evolution events in the *Fragaria nilgerrensis* genome. (a) Genome duplication in *F. nilgerrensis* (Fn)*, F*. *vesca* (Fv)*, P*. *avium* (Pa)*, R*. *chinensis* (Rc) and *R*. *occidentali* (Ro) genomes as revealed through 4DTv analyses. The percentages of the orthologous pairs (Fn vs Fv) between *F. nilgerrensis* (Fn) and *F*. *vesca* (Fv), the orthologous pairs (Fn vs Pa) between *F. nilgerrensis* (Fn) and *P*. *avium* (Pa), the orthologous pairs (Fn vs Rc) between *F. nilgerrensis* (Fn) and *R*. *chinensis* (Rc), the orthologous pairs (Fn vs Ro) between *F. nilgerrensis* (Fn) and *R*. *occidentali* (Ro), and paralogous gene pairs within the *F. nilgerrensis* (Fn vs Fn) genomes are plotted against their calculated 4DTv values; (b) distribution of synonymous substitution rates (Ks) for homologous gene groups. (c) Venn diagram represents the shared and unique gene families among *F. nilgerrensis*, *F*. *vesca*, *Prunus avium*, *Rosa chinensis* and *Rubus occidentalis.*

4DTv (fourfold synonymous third‐codon transversion) values among *F*. *nilgerrensis*, *F. vesca*, *P*. *avium*, *R*. *chinensis* and *R*. *occidentalis* were calculated using the orthologous gene pairs among these species. No obvious peak could be seen in the *F. nilgerrensis* and *F. vesca* paralog curves, while *P*. *avium*, *R*. *chinensis* and *R*. *occidentalis* had distinct peaks (Figure 2a). We also analysed the substitution rate (Ks) values in *F. nilgerrensis*, *F. vesca*, *P*. *avium*, *R*. *chinensis* and *R*. *occidentalis*. The peak Ks value was smaller than 0.1 for orthologous gene pairs between *F*. *nilgerrensis* and *F*. *vesca* (Figure 2b). The 4DTv and Ks results suggest that *F. nilgerrensis* genome did not occur an additional species‐specific whole‐genome duplication.

**Figure 3:**
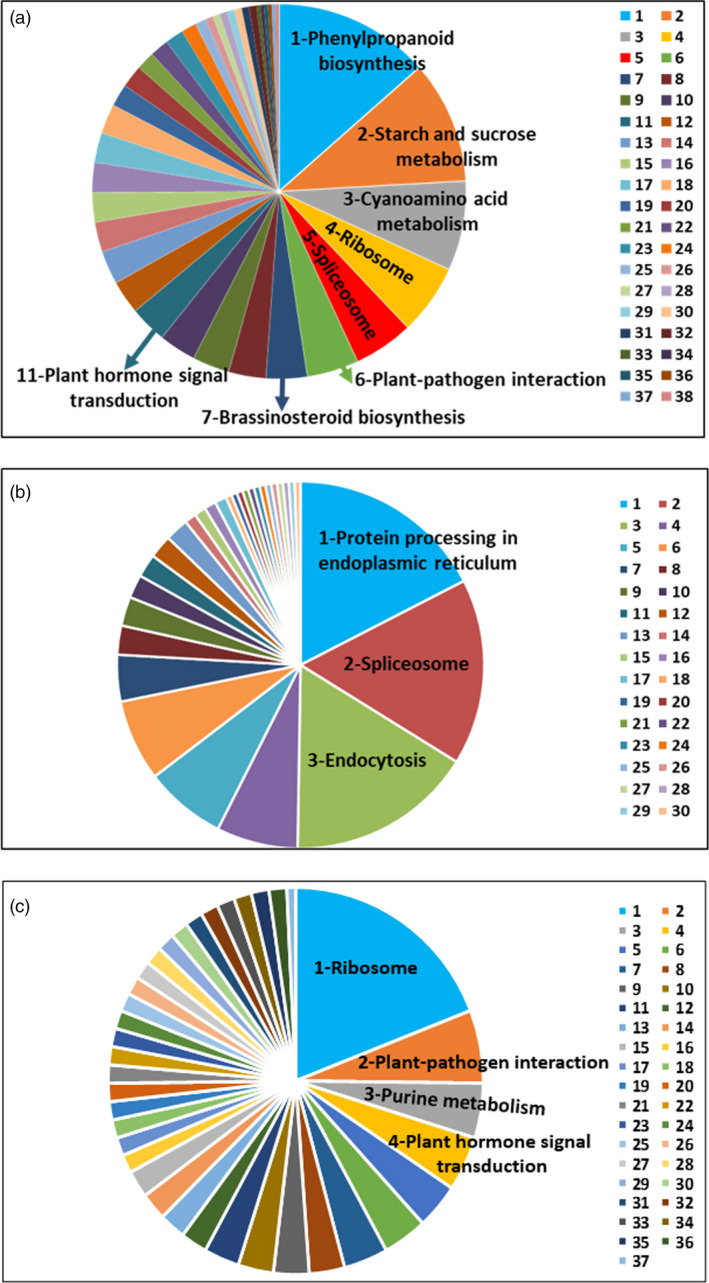
Functional classifications of expansion, contraction and unique genes in *Fragaria nilgerrensis* by KEGG. (a) Functional classification of expansion genes in *F. nilgerrensis*, 1. phenylpropanoid biosynthesis, 2. starch and sucrose metabolism, 3. cyanoamino acid metabolism, 4. ribosome, 5. spliceosome, 6. plant–pathogen interaction, 7. brassinosteroid biosynthesis, 8. galactose metabolism, 9. oxidative phosphorylation, 10. carbon metabolism, 11. plant hormone signal transduction, 12. pentose and glucuronate interconversions, 13. amino sugar and nucleotide sugar metabolism, 14. photosynthesis, 15. DNA replication, 16. nucleotide excision repair, 17. mismatch repair, 18. homologous recombination, 19. cysteine and methionine metabolism, 20. biosynthesis of amino acids, 21. pentose phosphate pathway, 22. glutathione metabolism, 23. sulphur metabolism, 24. RNA transport, 25. endocytosis, 26. purine metabolism, 27. pyrimidine metabolism, 28. inositol phosphate metabolism, 29. porphyrin and chlorophyll metabolism, 30. RNA polymerase, 31. phosphatidylinositol signalling system, 32. protein processing in endoplasmic reticulum, 33. glycine, serine and threonine metabolism, 34. monobactam biosynthesis, 35. lysine biosynthesis, 36. aminoacyl‐tRNA biosynthesis, 37. 2‐Oxocarboxylic acid metabolism, 38. ubiquitin mediated proteolysis；(b) Functional classification of contraction genes in *F. nilgerrensis,* 1. protein processing in endoplasmic reticulum, 2. spliceosome, 3. endocytosis, 4. pentose and glucuronate interconversions, 5. starch and sucrose metabolism, 6. linoleic acid metabolism, 7. alpha‐linolenic acid metabolism, 8. mRNA surveillance pathway, 9. plant hormone signal transduction, 10. tryptophan metabolism, 11. phenylpropanoid biosynthesis, 12. flavonoid biosynthesis, 13. stilbenoid, diarylheptanoid and gingerol biosynthesis, 14. steroid biosynthesis, 15. tyrosine metabolism, 16. isoquinoline alkaloid biosynthesis, 17. tropane, piperidine and pyridine alkaloid biosynthesis, 18. glycine, serine and threonine metabolism, 19. valine, leucine and isoleucine biosynthesis, 20. arginine and proline metabolism, 21. phenylalanine metabolism, 22. glutathione metabolism, 23. glycerophospholipid metabolism, 24. terpenoid backbone biosynthesis, 25. monoterpenoid biosynthesis, 26. carbon metabolism, 27. biosynthesis of amino acids, 28. ribosome biogenesis in eukaryotes, 29. protein export, 30. plant–pathogen interaction; (c) Functional classification of unique genes in *F. nilgerrensis*, 1. ribosome, 2. plant–pathogen interaction, 3. purine metabolism, 4. plant hormone signal transduction, 5. carbon metabolism, 6. ubiquitin mediated proteolysis, 7. phagosome, 8. citrate cycle, 9. pyrimidine metabolism, 10. RNA polymerase, 11. DNA replication, 12. glycolysis, 13. RNA transport, 14. SNARE interactions in vesicular transport, 15. endocytosis, 16. pentose and glucuronate interconversions, 17. oxidative phosphorylation, 18. photosynthesis, 19. lysine degradation, 20. tryptophan metabolism, 21. glutathione metabolism, 22. starch and sucrose metabolism, 23. brassinosteroid biosynthesis, 24. triterpenoid biosynthesis, 25. tropane, piperidine and pyridine alkaloid biosynthesis, 26. 2‐Oxocarboxylic acid metabolism, 27. biosynthesis of amino acids, 28. RNA degradation, 29. spliceosome, 30. proteasome, 31. nucleotide excision repair, 32. mismatch repair, 33. homologous recombination, 34. protein processing in endoplasmic reticulum, 35. peroxisome, 36. AGE‐RAGE signalling pathway in diabetic complications, 37. pentose phosphate pathway.

There were 362 and 211 gene families that expanded and contracted in the *F*. *nilgerrensis* genome, respectively, after divergence from *F. vesca*. Compared with *F*. *vesca*, the total numbers of significantly expanded families in *F*. *nilgerrensis* were much higher (362 for *F*. *nilgerrensis* compared with 146 for *F*. *vesca*), whereas the significantly contracted families in *F*. *nilgerrensis* (211) were less than those in *F*. *vesca* (474) (Figure 1a). We observed that 311 of the genes from expanded gene families were clustered in 38 KEGG pathways (Figure 3a). Among the expanded genes, genes involved in phenylpropanoid biosynthesis (42), starch and sucrose metabolism (33), and cyanoamino acid metabolism (24) represented the most abundant groups. In addition to the primary metabolic process, genes related to plant–pathogen interaction (14), brassinosteroid biosynthesis (11) and plant hormone signal transduction (10) also significantly expanded (Figure 3a; Appendix S2). A total of 195 genes in the contracted families were annotated to KEGG pathways. KEGG pathway analysis suggested that contracted gene families were mainly participated in endoplasmic reticulum (34), spliceosome (32) and endocytosis (32) (Figure 3b; Appendix S3). The functional classifications of genes involved in expanded and contracted gene families account for various traits of *F. nilgerrensis*, including its thick leaves, strong adaptability and disease resistance. Next, we compared the gene families among the five Rosaceae genomes (including *F*. *vesca*, *R*. *chinensis*, *P*. *avium* and *R*. *occidentalis*) and identified 17 034 gene families consisting of 25 669 genes in *F. nilgerrensis*. *F*. *nilgerrensis* shared the most gene families with the *F. vesca* and *R*. *chinensis* than *P*. *avium* and *R*. *occidentalis*. In addition, *F*. *nilgerrensis*, *F*. *vesca*, *R*. *chinensis*, *P*. *avium* and *R*. *occidentalis* had similar numbers of gene families, which further validates the accuracy and completeness of our gene predictions at the gene family level. Of these gene families, 11 591 were shared by all five genomes and 350 gene families, consisting of 1099 genes, were unique to *F*. *nilgerrensis* (Figure 2c). KEGG analysis showed the putative *F*. *nilgerrensis*‐specific genes were mainly involved in ribosome (25), plant–pathogen interaction (8), purine metabolism (6) and plant hormone signal transduction (6) (Figure 3c; Appendix S4).

### Repetitive sequences and transposable elements

**Figure 4:**
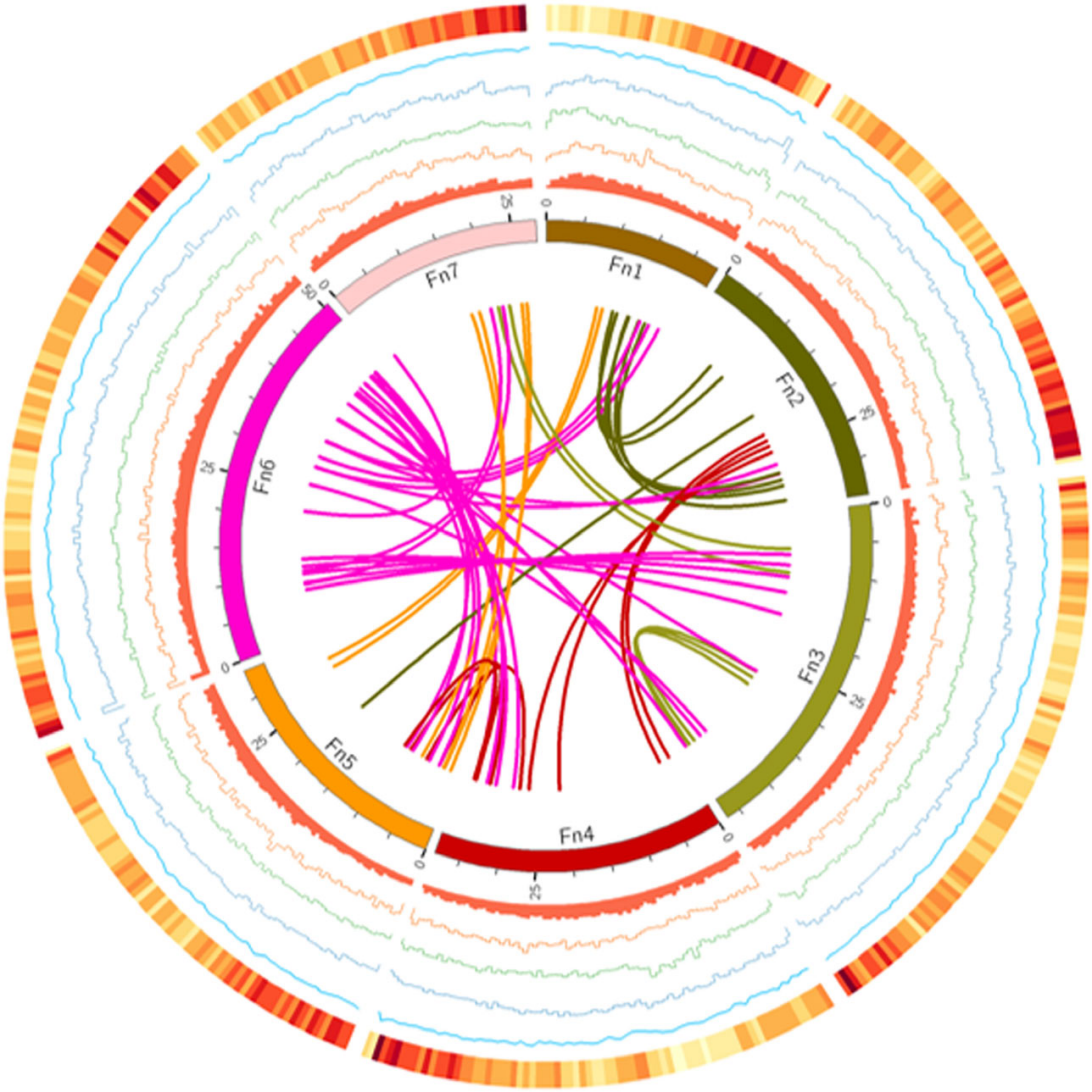
Genome landscape of *Fragaria nilgerrensis*. Elements are shown in the following scheme (from inner to outer). (i) Syntenic relationships among different chromosomes of *F. nilgerrensis*; (ii) distribution of repeats (window size, 100 kb); (iii) distribution of Copia elements (window size, 100 kb); (iv) distribution of Gpysy elements (window size, 100 kb); (v) distribution of TIR elements (window size, 100 kb); (vi) distribution of GC content (window size, 100 kb); (vii) gene density (window size, 100 kb).

To obtain a genome‐wide annotation of repetitive sequences of *F*. *nilgerrensis*, RepeatMasker package (Tarailo‐Graovac and Chen, 2009) was used to annotate repetitive sequence based on a combination of Repbase (Bao *et al.*, 2015) and the *de novo* predicted repeats database (see Methods for details). In the *F*. *nilgerrensis* genome, transposable elements (TEs) represented 117.22 Mb (43.36% of the 270.3 Mb final assembly; Table 2). The most abundant repetitive sequences in this genome were retrotransposons or class I elements (48.68% of TE content, 21.11% of the sequenced genome), of which long terminal repeat retrotransposons (LTR‐RTs) accounted for 16.55 % of the genome assembly (Table 2). The numbers of Gypsy elements were more than Copia elements (Figure 4; Table 2). Non‐LTR retrotransposons long intersperced nuclear elements (LINEs) and potential short intersperced nuclear elements (SINEs) represented 3.15 % of the sequence genome (Table 2). DNA transposons, also called class II elements (DNA transposons and Helitrons), maked up 39.49 % of the TE content (17.12% of the genome assembly; Table 2).

**Table 2:** The comparison of repetitive sequences between *Fragaria nilgerrensis and F*. *vesca* (Hawaii‐4)

Type	*F. nilgerrensis*			*F. vesca*		
	Number	Length (bp)	Rate (%)	Number	Length (bp)	Rate (%)
ClassI	77 778	57 063 567	21.11	78062	49 183 869	22.32
ClassI/DIRS	2172	1 968 677	0.73	1490	1 181 727	0.54
ClassI/LINE	19 088	8 315 089	3.08	12895	4 938 717	2.24
ClassI/LTR	353	265 376	0.10	382	292 959	0.13
ClassI/LTR/Copia	22 217	16 264 615	6.02	15433	12 945 703	5.87
ClassI/LTR/Gypsy	30 852	28 207 381	10.44	25009	21 674 646	9.84
ClassI/PLE|LARD	1620	1 242 370	0.46	20872	7 424 218	3.37
ClassI/SINE	863	195 484	0.07	1257	193 545	0.09
ClassI/SINE|TRIM	9	6858	0.00	0	0	0.00
ClassI/TRIM	422	514 143	0.19	598	418 479	0.19
ClassI/Unknown	182	95651	0.04	126	113 875	0.05
ClassII	73 218	46 284 298	17.12	42 842	22 754 418	10.33
ClassII/Crypton	1	36	0.00	2	152	0.00
ClassII/Helitron	7549	4 147 052	1.53	6401	2 833 166	1.29
ClassII/MITE	582	156 004	0.06	1834	439 172	0.20
ClassII/Maverick	171	133 086	0.05	332	135 320	0.06
ClassII/TIR	51 313	36 948 539	13.67	26 881	16 869 199	7.66
ClassII/Unknown	13 602	4 910 105	1.82	7392	2 477 409	1.12
PotentialHostGene	9641	4 062 514	1.50	1782	625 063	0.28
SSR	142	25707	0.01	150	140 915	0.06
Unknown	28 203	9 884 875	3.66	13 627	4 101 107	1.86
Total	160 779	117 216 089	43.36	122 836	76 805 372	34.85

Next, we analysed TE differences between *F*. *nilgerrensis* and *F*. *vesca*. Approximately 117.22 Mb and 76.81 Mb TEs were identified in the *F*. *nilgerrensis* and *F*. *vesca* genomes, respectively (Table 2). We found that retrotransposon elements accounted for the largest TEs genomic fraction in *F*. *vesca* (22.32%), similar to *F*. *nilgerrensis* (21.11%). More copies for class II elements or DNA transposons were found in *F*. *nilgerrensis* than *F*. *vesca*, especially in TIR (Figure 4; Table 2). TIR accounted for 13.67% of the genome assembly in *F*. *nilgerrensis*, whereas TIR represented 7.65 % of the genome assembly in *F*. *vesca*. This suggests that the difference in genome size between *F*. *nilgerrensis* and *F*. *vesca* is mainly due to an expansion of the TEs and the difference in the repeats content (~40 Mb) can account for 80.98 % of the increased genome size in *F*. *nilgerrensis* compared with *F*. *vesca* genome.

**Figure 5:**
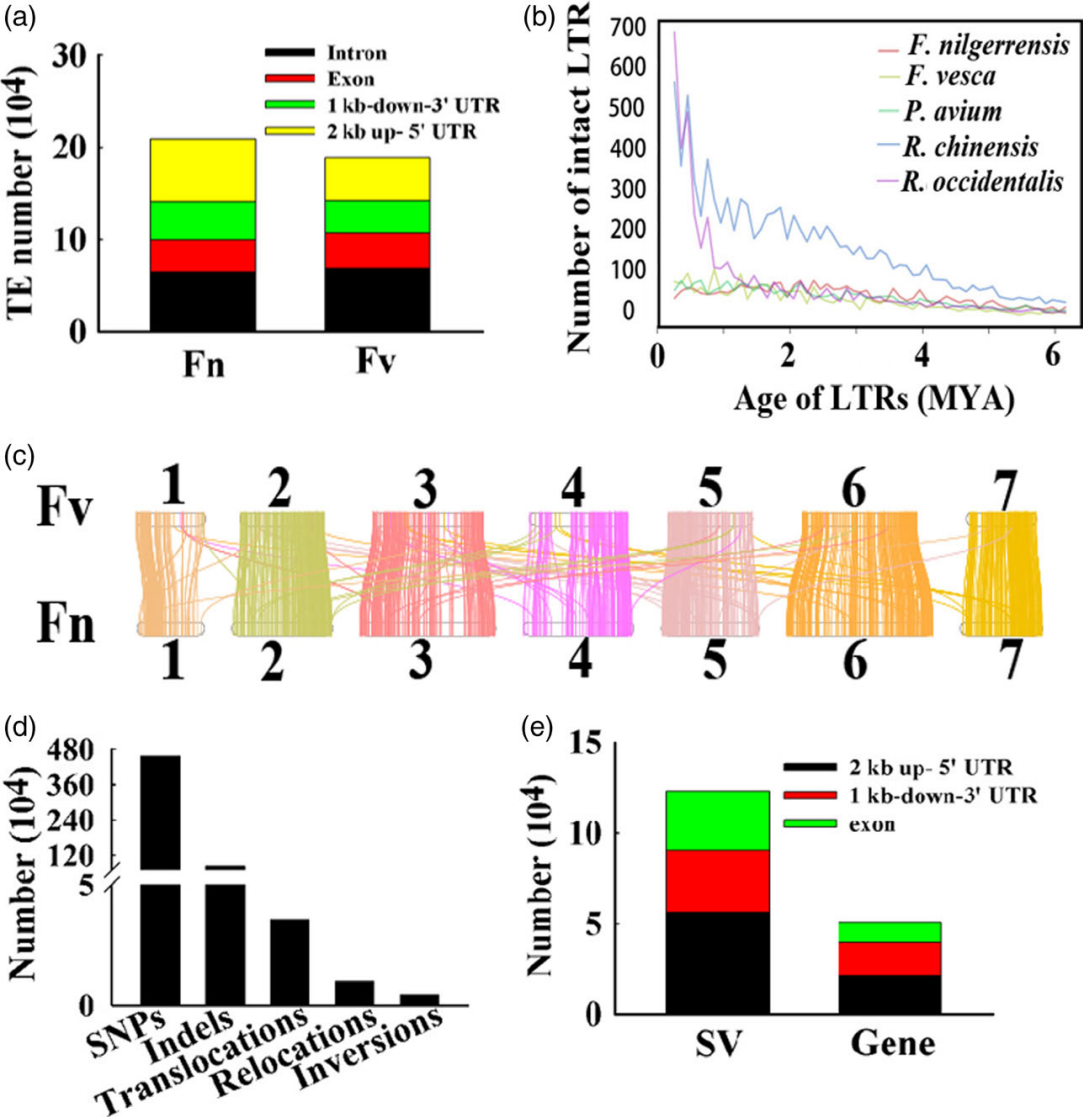
The comparison of *Fragaria nilgerrensis* and *F*. *vesca* genomes. (a) The distribution of transposable element (TE) in the exon, promoter (2kb up‐ 5' UTR) and downstream (1kb‐down‐3' UTR) regions of genes in the *F*. *nilgerrensis* and *F*. *vesca* genome. (b) Distribution of insertion ages of LTR retrotransposons. The *x*‐axis represents the estimated insertion age of the LTR retrotransposons. The y‐axis represents the number of intact LTR retrotransposons. (c) Syntenic blocks share between the *F. nilgerrensis* and *F*. *vesca* genomes. (d) The numbers of SNPs, Indels, inversions, translocations, relocations in *F*. *nilgerrensis* genome compared with *F*. *vesca* genome. (e) The distribution of structural variation (SV) in the exon, promoter (2kb up‐ 5' UTR) and downstream (1kb‐down‐3' UTR) regions of genes in the *F*. *nilgerrensis* genome.

We also compared the distribution of TEs in 16 820 orthologous gene pairs between *F. nilgerrensis* and *F. vesca*. We found the numbers of TEs distributed in promoters and downstream regions of genes were similar between *F*. *nilgerrensis* and *F*. *vesca*. The number of TEs in intron regions of *F*. *nilgerrensis* was significantly larger than *F*. *vesca* (Figure 5a). Interestingly, TEs were also highly enriched in exon regions of genes in the *F*. *nilgerrensis* compared with the corresponding regions of *F*. *vesca* (Figure 5a).

Finally, we investigated insertion ages for LTR retrotransposons among *F*. *nilgerrensis*, *F*. *vesca*, *R*. *chinensis*, *P*. *avium* and *R*. *occidentalis*. The insertion ages of LTR retrotransposons displayed similar insertion profiles among the genomes of *F*. *nilgerrensis*, *F*. *vesca* and *P*. *avium* (Figure 5b). *R*. *chinensis* and *R*. *occidentalis* had more LTR retrotransposons than *F*. *nilgerrensis* and carried the largest number of LTR retrotransposons with insertion ages less than 0.2 Mya (Figure 5b), which might result from variable environmental conditions.

### ***Genome comparison between F*** *.* ***nilgerrensis and F*** *.* ***vesca***

Genetic variations, such as insertion and deletion polymorphisms are responsible for genetic diversity and phenotypic variations (Zhang *et al.*, 2019). *F*. *nilgerrensis* has obvious phenotypic differences with respect to *F*. *vesca*, which made us comprehensive catalogues of genomic variations between these two species. Using a full‐genome alignment approach, we identified 403 syntenic blocks with 16 820 conserved genes covering 61.29% and 58.83% of the *F*. *nilgerrensis* and *F. vesca* genomes (Figure 5c), respectively. The large number of collinear blocks indicates good gene collinearity between the genomes of *F*. *nilgerrensis* and *F*. *vesca* (Figure 5c). We identified 4 561 825 SNPs in *F*. *nilgerrensis* genome compared with *F*. *vesca* genome and *F. nilgerrensis* genome had an average density of 16.9 SNPs per kilobase (Figure 5d). Among these SNPs, the G to A (15.18%), T to C (15.10%), A to G (15.07%) and C to T (15.17%) represented the most abundant groups (Table S7). Chromosome 6 had the highest number of SNPs (865 301), while chromosome 1 had the lowest number of SNPs (435 611; Table S8). In addition, we identified 846 301 Indels in *F*. *nilgerrensis* genome compared with *F*. *vesca* genome and *F. nilgerrensis* genome had an average density of 3.1 Indels per kilobase (Figure 5d). Chromosome 6 had the highest number of SNPs and Indels, whereas chromosome 1 had the lowest number of SNPs and Indels (Table S8–S9). The numbers of SNPs and Indels on chromosomes were consistent with the length of corresponding chromosome (Table 1; Table S8–S9). Additionally, we also identified 4243 inversions, 35 498 translocations and 10 099 relocations in *F*. *nilgerrensis* genome relatively to *F*. *vesca* genome (Figure 5d). Next, the distribution of structural variation (SV) in the exon, promoter (2kb up‐ 5' UTR) and downstream (1kb‐down‐3' UTR) regions of genes in the *F*. *nilgerrensis* genome was carried out. We found 32 930 SV in the exon regions of 10 946 genes, 56 146 SV in the promoter regions of 21 369 genes, and 33 886 SV in the downstream regions of 18 300 genes (Figure 5e). The great variations between *F*. *nilgerrensis* and *F*. *vesca* may provide some information for their phenotypic differences.

### ***Lower expression of FnMYB10 may lead to white‐fruited F*** *.* ***nilgerrensis***

**Figure 6:**
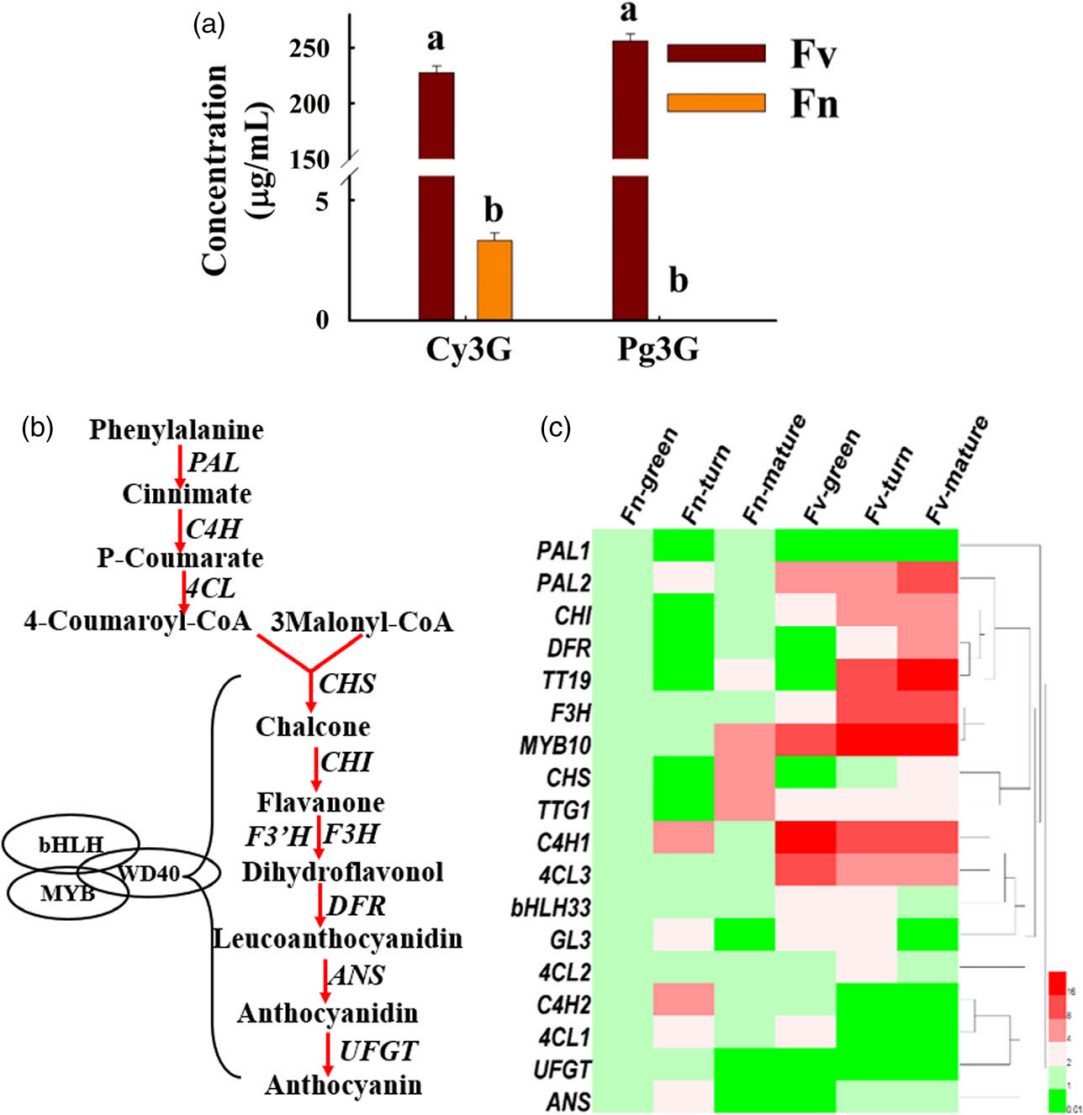
The anthocyanin content and anthocyanin‐related gene expression between *Fragaria nilgerrensis* (Fn) and *F*. *vesca* (Fv). (a) The anthocyanin content and compositions between the mature fruits of *F*. *nilgerrensis* (Fn) and *F*. *vesca* (Fv). (b) Simplified scheme of anthocyanin biosynthetic and regulatory pathway in plants. Biosynthetic genes are shown in right or left side of arrow and the transcription factors are shown in oval. *PAL* phenylalanine ammonia lyase, *C4H* cinnamate‐4‐hydroxylase, *4CL* 4‐coumarate CoA ligase, *CHS* chalcone synthase, *CHI* chalcone isomerase, *F3H* flavanone 3‐hydroxylase, *F3’H* flavonoid 3’‐hydroxylase, *DFR* dihydroflavonol 4‐reductase, *ANS* anthocyanidin synthase*, LDOX*, leucoanthocyanidin dioxygenase, *UFGT*, UDP‐flavonoid glucosyl transferase. (c) The gene expression heatmap of anthocyanin‐related genes in three developmental stages (green, turn and ripe stages) of white‐fruited *F*. *nilgerrensis* (Fn) and red‐fruited *F*. *vesca* (Fv). Heml software is used for making gene expression heatmap.

The colour of strawberry mainly results from the accumulation of anthocyanin and the fruits of *F*. *nilgerrensis* are white. The mature red fruits of *F*. *vesca* (Ruegen) mainly accumulate cyanidin 3‐glucoside (Cy3G) and pelargonidin 3‐glucoside (Pg3G), while *F*. *nilgerrensis* only has very little accumulated cyanidin 3‐glucoside in its mature white fruits (Figure 6a; Figure S9). The anthocyanin content was dramatically reduced in *F*. *nilgerrensis* mature fruits compared with red fruits *F*. *vesca*. To identify candidate genes that might explain the loss‐of‐colour phenotype in white‐fruited *F*. *nilgerrensis*, we compared the expression of anthocyanin‐related genes in three developmental stages (green, turn and ripe stages) of white‐fruited *F*. *nilgerrensis* with red‐fruited *F*. *vesca* by qRT‐PCR to test whether gene expressions of some of anthocyanin‐related genes are blocked in *F*. *nilgerrensis*. We found transcript levels of most of anthocyanin biosynthesis pathway genes (Figure 6b), such as *PAL1.2*, *C4H1*, *4CL3*, *CHI*, *F3H* and *DFR*, were significantly down‐regulated in three developmental stages in *F*. *nilgerrensis* compared with *F. vesca* (Figure 6c)*.* Interestingly, we found *MYB10*, a key positive factor in regulating anthocyanin accumulation, was also dramatically reduced in three developmental stages in *F*. *nilgerrensis* relatively to *F. vesca* (Figure 6c). The lower expression of *FnMYB10* may be responsible for the white fruit phenotype of *F*. *nilgerrensis*.

**Figure 7:**
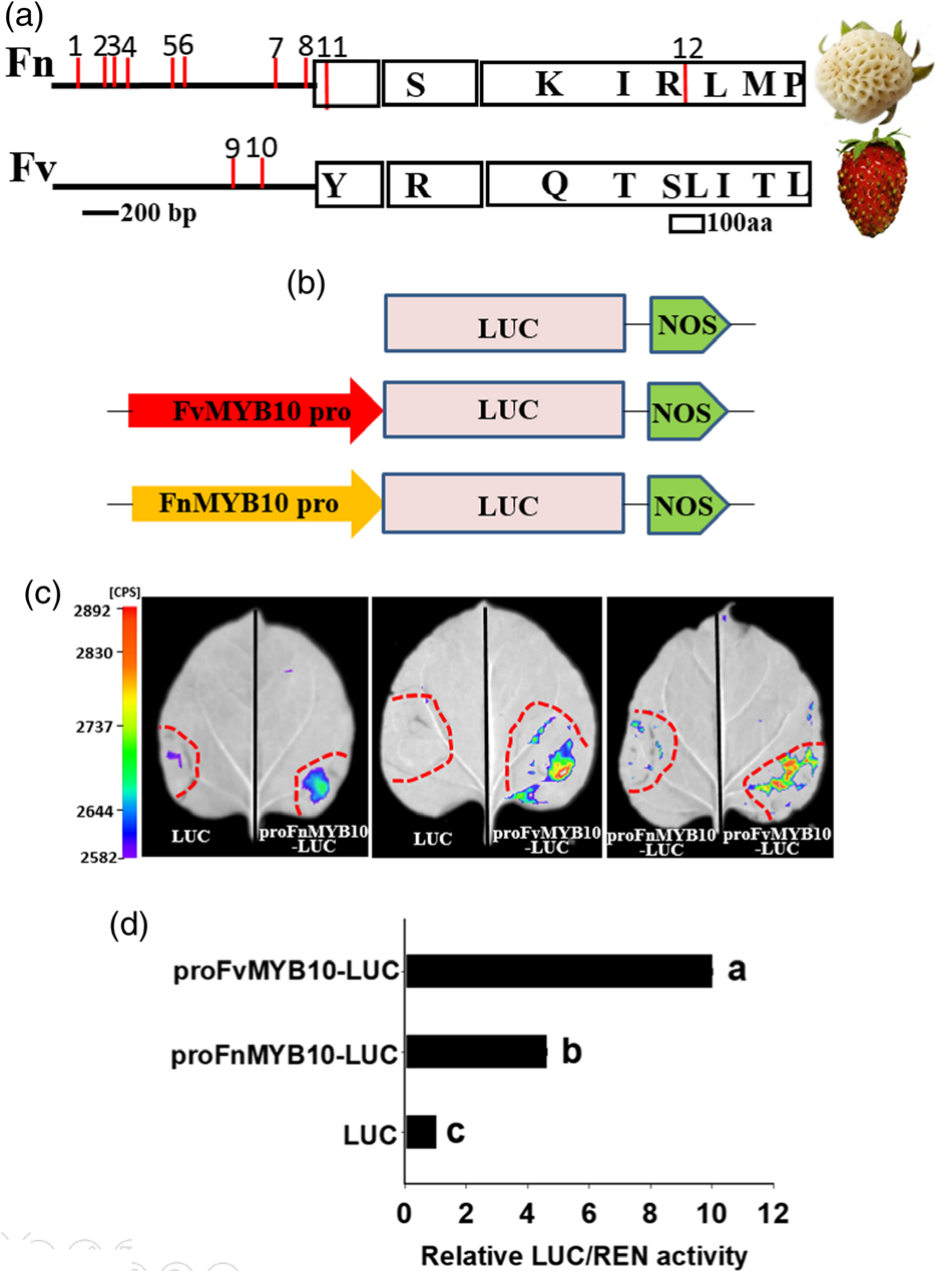
The comparison of sequences and transcription activity of *MYB10* promoter between *Fragaria nilgerrensis* (Fn) and *F*. *vesca* (Fv). (a) The comparison of MYB10 promoter and protein between *F*. *nilgerrensis* (Fn) and *F*. *vesca* (Fv). Promoter and protein are shown as lines and boxes, respectively. Red lines (1–8) represent missing cis‐acting elements in the promoter of *F*. *nilgerrensis* compared with *F*. *vesca*. Red lines (9, 10) represent missing cis‐acting elements in the promoter of *F*. *vesca* compared with *F*. *nilgerrensis*. Red lines (11, 12) represent missing amino acids in *F*. *nilgerrensis* compared with *F*. *vesca*. Amino acids changes between *F*. *nilgerrensis* (Fn) and *F*. *vesca* (Fv) are shown in boxes. (b) Schematic diagrams of the Luc, ProFnMYB10‐Luc and ProFvMYB10‐Luc reporter constructs used for tobacco transient expression assay. (c) Luciferase activity assay. The Luc, ProFnMYB10‐Luc and ProFvMYB10‐Luc are transformed into *Nicotiana benthamiana*. (d) The comparison of luciferase activity. The transcriptional activity of these infiltrated tobacco leaves based on the ratio of LUC to REN is investigated by Dual Luciferase Reporter Gene assay kit. Different letters indicate significant differences (*P* < 0.05, based on Duncan’s multiple range test).

We cloned and sequenced the coding regions of *MYB10* from white‐fruited *F. nilgerrensis* (*FnMYB10*) and red‐fruited *F. vesca* (*FvMYB10*). *FnMYB10* contains an open reading frame (ORF) of 702 bp that encodes a protein of 233 amino acids and the ORF of *FvMYB10* is 708 bp which encodes a protein of 235 amino acids (Figure S10). Alignment of the amino acids of FnMYB10 and FvMYB10 displayed that there were two amino acid deletion and six amino acid differences with respect to FvMYB10 (Figure 7a; S10). The transcript level of *FnMYB10* was significantly reduced in the white‐fruited *F*. *nilgerrensis* and the FnMYB10 amino acids did not result in frameshift and premature stop codon (Figure 7a; S10). Thus, we hypothesized that the mutation in the promoter region might lead to loss‐of‐colour phenotype in white‐fruited *F*. *nilgerrensis*. We first use genome sequence information to analyse the promoter of *MYB10* and found they were some variations in the promoter between *F*. *nilgerrensis* and *F*. *vesca*. Next, we amplified the *MYB10* promoter from *F*. *nilgerrensis* and *F*. *vesca* by PCR and the primer sets were shown in Table S10. The promoter sequences between the *FnMYB10* and *FvMYB10* showed that there were eight deletions and two insertions (nucleotide changes more than five were shown; Figure 7a; S11). We identified and predicted the missing cis‐acting elements in the promoter region of *FnMYB10* through aligning to PLACE database (Higo *et al.*, 1999) and found some missing cis‐acting elements in *F*. *nilgerrensis* were involved in sugar responses (Sun *et al.*, 2003) and light regulation (Terzaghi and Cashmore, 1995). To investigate whether the promoter sequence differences in *MYB10* affect *MYB10* promoter activity, promoters from *FnMYB10* and *FvMYB10* were fused to luciferase gene (Figure 7b) and transformed into tobacco leaves. We investigated the luciferase activity of ProFnMYB10‐Luc and ProFvMYB10‐Luc in different tobacco leaves and different parts of the same tobacco leaf (Figure 7c). Interestingly, both of them showed the ProFvMYB10‐Luc exhibited significantly higher Luc activity than the ProFnMYB10‐Luc (Figure 7c,d). These results indicate that the variations at the upstream regulatory region of *FnMYB10* reduce the promoter activity of the *FnMYB10* gene and result in its lower expression, which may be responsible for its white fruit phenotype of *F*. *nilgerrensis*.

## Discussion

There are 24 wild species of *Fragaria* with different ploidy have been identified (Folta and Davis, 2006; Hummer and Hancock, 2009; Staudt, 2009). These wild *Fragaria* species are a valuable resource for genetic evolution and comparative genomics research. Additionally, wild species of *Fragaria* have many important characteristics, which can be used for substantial improvements of the cultivated strawberry. However, the genome information of most of these wild *Fragaria* species is largely unavailable and it is difficult to obtain the important trait from wild species without the availability of high‐quality reference genome. The genome of *F*. *vesca* (Hawaii‐4) is assembled by Shulaev *et al* *. * (2011) using a combination of different short‐read technologies and the contig N50 length is only about 27 kb, which is incomplete and highly fragmented. Next, some studies re‐annotate *F*. *vesca* genome based on Illumina‐based (Darwish *et al.*, 2015) and the mixture of Illumina‐ and SMRT‐based RNA‐seq transcriptome sequences (Li *et al.*, 2017). Edger et al. (2017 yields a high‐quality reference genome of *F*. *vesca* with a contig N50 length of around 7.9 Mb based on a combination of single‐molecule sequencing and optical mapping, which dramatically improved the genome quality compared with the short‐read‐based draft genome of *F*. *vesca*. The genome sequences of three diploid wild species, *F*. *iinumae*, *F*. *nipponica*, *F*. *nubicola*, and one tetraploid wild species *F*. *orientalis* are obtained using Illumina and Roche 454 platforms (Hirakawa *et al.*, 2014). These draft genomes are based on second‐generation sequencing technologies which are difficult to get the large repetitive sequences and lead to highly fragmented genome assemblies (Michael and VanBuren, 2015). Transcriptome sequences of four diploid wild species *F*. *nilgerrensis*, *F*. *pentaphylla*, *F*. *mandshurica*, *F*. *viridis*, and three tetraploid wild species *F*. *corymbosa*, *F*. *moupinensis* and *F*. *tibetica* are released by Qiao *et al* *. * (2016) for evolutionary patterns analysis, while the genome sequences of these wild species of *Fragaria* are not available. Here, we provide a high‐quality genome of diploid strawberry *F*. *nilgerrensis* using SMRT Pacific Biosciences sequencing and Hi‐C genome scaffolding. A high‐quality reference genome is critical for obtaining structural variants, linking phenotype–genotype associations, using for genome evolution and comparative genomics research and identifying gene controlling important traits (Schulz *et al.*, 2013; Song *et al.*, 2018; Zhang *et al.*, 2019).

The genome size of *F*. *nilgerrensis* is 270.3 Mb. The flow cytometric analysis of *F*. *nilgerrensis* suggests that the estimated *F*. *nilgerrensis* genome size is 1C = 274±3.80 Mb (Chen *et al.*, 2015), which is very close to our assembly size (270.3 Mb). The genome size of *F*. *vesca* V4 genome is 220.4 Mb which is 49.9 M smaller than *F*. *nilgerrensis*. The major reason is dramatic expansion of TEs in *F*. *nilgerrensis*, especially in ClassII/TIR, ClassI/LTR/Copia and ClassI/LTR/Gypsy (Table 2). Strong correlation between genome size and the proportion of TEs has been reported in many studies (Biemont, 2008; Huang *et al.*, 2019; Tenaillon *et al.*, 2011). The contig N50 size of *F*. *nilgerrensis* is ∼8.5 Mb which is larger than *F*. *vesca* V4 genome with an N50 length of∼7.9 Mb and less than model plants Arabidopsis with an N50 length of 12.3 Mb (Michael *et al.*, 2018). The smaller genome size and sufficient genetic resources may account for the much larger contig N50 in Arabidopsis than *F*. *nilgerrensis*. We identified 28 780 genes in *F*. *nilgerrensis* and the numbers of genes of *F. nilgerrensis* are similar to *F. vesca* V4 genome. However, the predicted genes in *F*. *nilgerrensis* genome are significantly less than the results of Qiao *et al *(2016). They annotate 82537 genes in *F*. *nilgerrensis* based on RNA‐seq data and the reference genome of *F*. *nilgerrensis* is not available at that time, which may result in incorrect size genes, mis‐annotated intron/exon junctions and mis‐annotated transcripts (Darwish *et al.*, 2015). This phenomenon also shows in woodland strawberry *F*. *vesca* (Edger et al. 2017; Shulaev *et al.*, 2011). A total of 34 809 genes are identified in *F. vesca* V1 genome utilizing second‐generation sequencing technologies, while the numbers of genes are reduced to 28 588 in *F*. *vesca* V4 genome using single‐molecule real‐time sequencing. Therefore, a high‐quality genome is also invaluable for gene identification and annotation.

Phylogenetic analysis of the nuclear internal transcribed spacer region and chloroplast DNA sequences suggests that *F*. *nilgerrensis*, *F*. *nipponica*, *F*. *daltoniana*, *F*. *pentaphylla* and *F*. *gracilis* are in the some clade and *F*. *nilgerrensis* appears as a high divergence evolutionary unit within this clade (Potter *et al.*, 2000). *F*. *vesca*, *F*. *nubicola* and the polyploid wild species are in other major clade (Potter *et al.*, 2000). The cross compatibility of *F*. *vesca* to many *Fragaria* species is comparatively high (Hancock and Luby, 1993; Sargent *et al.*, 2004), while *F. nilgerrensis* has a comparatively low compatibility to other *Fragaria* species (Hancock and Luby, 1993). Many studies also show that phylogenetic relationship between *F*. *nilgerrensis* and *F*. *vesca* is distant (Liu *et al.*, 2016; Njuguna *et al.*, 2013; Potter *et al.*, 2000; Qiao *et al.*, 2016; Rousseau‐Gueutin et al. 2009). We actually found many differences between *F*. *nilgerrensis* and *F*. *vesca* on TEs, SNPs, Indels and SVs. TEs can replicate and integrate into different positions of the genome in changing environment, which are important for genetic diversity, phenotypic variation and adaptation (Niu *et al.*, 2019). The content and distributions of TEs were different between *F*. *nilgerrensis* and *F*. *vesca*. And *F*. *nilgerrensis* had more TEs than *F*. *vesca* and TEs were enriched in exon regions of genes in the *F. nilgerrensis* than the corresponding regions of *F*. *vesca*, suggesting that TEs could potentially contribute to the diversification of gene function in *F*. *nilgerrensis* (Casacuberta and Gonzalez, 2013; Chuong *et al.*, 2017; Li *et al.*, 2018). Genetic variations are an important factor for genetic diversity and phenotypic variations (Zhang *et al.*, 2019). The variations between *F*. *nilgerrensis* and *F*. *vesca* may result in phenotype differences between these two species. The phenotypic variations between ‘Golden Delicious’ genome and ‘Hanfu’ genome also suggest that genome variations account for different phenotypes between these two apple cultivars (Daccord *et al*, 2017; Zhang *et al*, 2019). In addition, some literatures show that *F*. *nilgerrensis* shares more common features with tetraploid *F*. *moupinensis* (Darrow, 1966), hexaploid *F*. *moschata* (Chambers et al. 2013) and decaploid *F*. *iturupensis* (Liston *et al.*, 2014). The availability of *F*. *nilgerrensis* genome may provide some information on the relationships among these species.

The distribution of *F*. *nilgerrensis* is in roadside, grassland and hillside areas in Yunnan, China (Figure S1) (Wang *et al.*, 2017), and *F*. *nilgerrensis* has many traits to adapt to changing environments including leathery and thick leaves, waterlogging tolerance and resistance to leaf disease (Guo *et al.*, 2017). Our results indicate that the significantly expanded families in *F*. *nilgerrensis* are mainly involved in phenylpropanoid biosynthesis, starch and sucrose metabolism, cyanoamino acid metabolism, plant–pathogen interaction, brassinosteroid biosynthesis, and plant hormone signal transduction compared with other species (Figure 3a). The expanded gene families in *F*. *nilgerrensis* may account for its phenotype and resistance to biotic/abiotic stress. Multiple studies have reported that the expanded families in different plants may contribute to these specific phenotype and abiotic and biotic stress tolerance, such as durian (Liu *et al.*, 2019; Teh *et al.*, 2017), wild barley (Liu *et al.*, 2019) and wild pear (Dong *et al.*, 2019). We also found 350 gene families (1099 genes) were unique to *F*. *nilgerrensis* compared with the other four Rosaceae species. *F*. *nilgerrensis*‐specific genes were enriched in pathways participated in ribosome, plant–pathogen interaction, purine metabolism and plant hormone signal transduction (Figure 3c). Specific genes related to development or defence responses are also reported in wild barley (Liu *et al.*, 2019) and orchardgrass genome (Huang *et al.*, 2019). The genotype‐specific genes are considered to be the existence of variable genomes (Amiri *et al.*, 2003; Dong *et al.*, 2019; Liu *et al.*, 2019; Teh *et al.*, 2017), and these genes may have significant roles in species‐specific biology and biological evolution (Xu *et al.*, 2015). Therefore, the potential of using the wild *Fragaria* species in breeding and identification of genes for agronomically important traits is available (Bors and Sullivan, 1998).

Fruit colour is an important trait for many horticulture crops and strawberry mainly accumulates anthocyanin in fruits. In this study, we found MYB10 might be responsible for the white fruit phenotype of *F*. *nilgerrensis*. A recent study shows a premature stop codon in *FaMYB10* in octoploid strawberry (*Fragaria* × *ananassa* Duch.) results in loss of red colour in white octoploid strawberry (Wang *et al.*, 2019). However, the coding regions of *FnMYB10* in *F*. *nilgerrensis* encoded functional proteins. Thus, the sequence variations in the upstream regulatory region of *FnMYB10* are likely responsible for lower expression of *FnMYB10*. MYB10 is considered as the master regulator in controlling anthocyanin accumulation in apple (Ban *et al.*, 2007; Chagne *et al.*, 2007; El‐Sharkawy *et al.*, 2015; Espley *et al.*, 2009; Espley *et al.*, 2007; Medina‐Puche *et al.*, 2015), pear (Wang *et al.*, 2013; Zhai *et al.*, 2015) and strawberry (Lin‐Wang *et al.*, 2014; Zhang *et al.*, 2017). The pattern of anthocyanin accumulation in many horticulture crops arises from the sequence variations or methylation in the promoter region of *MYB10* (Espley *et al.*, 2009; Medina‐Puche *et al.*, 2015; Wang *et al.*, 2013). Here, we also found there were many sequence variations in the promoter region of *F*. *nilgerrensis* compared with *FvMYB10* from red‐fruited *F. vesca* (Figure 7a; Figure S11). Among the sequence variations, we found two important cis‐acting elements involved in sugar responses (Sun *et al.*, 2003) and light regulation (Terzaghi and Cashmore, 1995) were missing in *F*. *nilgerrensis* promoter region compared with *F. vesca.* Sucrose is a crucial signal in promoting anthocyanin accumulation and affecting fruit ripening in strawberry (Jia *et al.*, 2013; Luo *et al.*, 2019). Light can regulate anthocyanin biosynthesis through activation of *FaMYB10* expression during strawberry fruit ripening (Kadomura‐Ishikawa *et al.*, 2014). Therefore, the missing of these two important cis‐acting elements in *MYB10* of *F. nilgerrensis* may affect the regulatory roles of sucrose and light in *MYB10.* Interestingly, the promoter from *F. nilgerrensis* and *F. vesca* showed different capacity in regulating luciferase gene expression and the luciferase activity of *F. nilgerrensis* promoter was significantly reduced compared with *F. vesca* (Figure 7c and d). Thus, the variations in the promoter of *FnMYB10* resulted in lower gene expression of *FnMYB10* to confer the loss‐of‐colour phenotypic change in *F. nilgerrensis*. These results will facilitate developing marker for fruit colour selection at an early stage and enrich the regulatory network of anthocyanin accumulation in strawberry.

Together, this genome of *F. nilgerrensis* will lay a solid foundation for conducting comparative genomics research, elucidating the relationships among different wild species of *Fragaria*, and identifying genes governing important agronomic traits.

## Experimental procedures

### Plant materials

The diploid *Fragaria nilgerrensis* (2*n* = 2*x* = 14) was used for genome sequencing. *F*. *nilgerrensis* was originally obtained from Zhongdian, Yunnan, China (altitude: 3321 m, 27°40′07″E, 99°43′17″N), and is maintained at Shenyang Agriculture University (123°33′58″E, 41°49′9″N; Shenyang). The fruit of *F*. *nilgerrensis* is white and the fruit of *F*. *vesca* accession ‘Ruegen’ is red. For the different ripening stages of *F*. *nilgerrensis* and *F*. *vesca*, fruits at 8 days (green stage), 24 days (turning stage) and 32 days (mature stage) after pollination were collected for qRT‐PCR analysis. In each ripening stage (8 days post‐anthesis, 24 days post‐anthesis and 32 days post‐anthesis), we profiled twenty fruits from twenty plants for qRT‐PCR. Mature fruits (32 days post‐anthesis) of *F*. *nilgerrensis* and *F*. *vesca* were used for testing the anthocyanin content and composition by HPLC.

### Illumina short‐read sequencing

Genomic DNA of *F*. *nilgerrensis* was extracted from young leaf tissue using a DNAsecure Plant Kit (TIANGEN, Beijing, China). The DNA quality and concentration were tested by 1% agarose gel electrophoresis and Qubit 2.0 Fluorometer (Life Technologies, Carlsbad, CA, USA). The 220‐bp paired‐end (PE) libraries were constructed using the NEBNext® Ultra™ DNA Library Prep Kit and sequenced on the Illumina HiSeq X Ten platform at the Biomarker Technologies Corporation, Beijing. The raw Illumina sequencing reads were processed with Trimmomatic program version 0.33 (Bolger *et al.*, 2014) and Cutadapt program version 1.13 (Martin, 2011) to remove adapters, leading and trailing ambiguous or low‐quality bases. Finally, 19.62‐Gb clean reads were used for the assembly evaluation and error correction of genome assembly.

### Genome size and heterozygosity analysis

We estimated *F*. *nilgerrensis* genome size and heterozygosity based on k‐mer analysis. 19.62‐Gb high‐quality sequencing reads were used to generate a k‐mer (k = 19) depth distribution curve using ‘kmer freq stat’ software developed by Biomarker Technologies Corporation (Beijing, China). The genome size (GS) of *F*. *nilgerrensis* was estimated by the following formula: GS = k‐mer number/average k‐mer depth (Gao et al. 2018). The highest peak in the k‐mer distribution was at the k‐mer depth of 57, with a k‐mer number of 17 016 192 556 and the depth of k‐mer more than 115 was the repetitive sequences (Figure S4). The heterozygosity was estimated at ∼0.1 % based on the formula described by Liu *et al* *. * (2013).

### PacBio SMRT sequencing and assembly

The SMRT Bell library was prepared using a DNA Template Prep Kit 1.0, and 20‐kb SMRTbell libraries were constructed. *De novo* assembly was carried out using Canu version 1.5 (Koren *et al.*, 2017), with the parameters ‘genomeSize = 250000000, and ‘corOutCoverage = 50’ and Falcon version 0.3.0 (https://github.com/PacificBiosciences/FALCON) with the parameters ‘length_cutoff = 3000, length_cutoff_pr = 8000’, and other parameters ‘default’. Quickmerge (Chakraborty *et al.*, 2016) was used for merging Canu and Falcon pipeline assemble results to produce a more contiguous assembly. Then, the Illumina data were aligned to the assembly contigs from Quickmerge pipeline using bwa mem v0.7.12 (Li, 2013). Finally, the draft assembly was corrected using Pilon version 1.22 (Walker *et al.*, 2014).

### Hi‐C assembly

To anchor scaffolds onto the chromosome, we constructed Hi‐C library by the Illumina HiSeq X Ten platform. The trimmed reads of the *F*. *nilgerrensis* genome were mapped to the assembly using BWA version 0.7.12 software (Li, 2013) with the parameters (bwa index ‐a bwtsw fasta; bwa aln ‐M 3 ‐O 11 ‐E 4 ‐t 2 fq1; bwa aln ‐M 3 ‐O 11 ‐E 4 ‐t 2 fq2). HiC‐Pro pipeline (Servant *et al.*, 2015) was used for filtering low‐quality reads and quality assessment. The parameters of HiC‐Pro were mapped 2hic fragments.py ‐v ‐S ‐s 100 ‐l 1000 ‐a ‐f ‐r ‐o. LACHESIS (Hariharan and Toyama, 2004) was used for parsing and modelling location of genome sequence with the following parameters: cluster min re sites = 48; cluster max link density = 2, cluster noninformative ratio = 2, order min n res in trun = 14, order min n res in shreds = 15). Finally, PBjelly (English *et al.*, 2012) was used for gap filling for LACHESIS‐based assembly to improve the accuracy and completeness of the genome assembly.

### RNA sequencing

RNA was extracted from five tissues (young leaves, stems, flowers, mature fruits and roots) of the same plant used for RNA sequencing (RNA‐Seq) according to the Easy Spin RNA extraction kit (Sangon Biotech, Shanghai, China). The concentration of RNA sample was tested by a NanoDrop spectrophotometer (Thermo Fisher Scientific Inc., Waltham, MA, USA) and a Qubit 2.0 Fluorometer (Life Technologies). The integrity of RNA sample was tested via a Bioanalyzer 2100 (Agilent Technologies, Santa Clara, CA). One RNA library with an average insert size of 250–300 bp was produced using the NEBNext UltraTM RNA Library Prep Kit following descriptions by the manufacturer, Illumina (NEB, Beverly, MA, USA). Library quality was detected on the Agilent Bioanalyzer 2100 system. A combined library of young leaves, stems, flowers, mature fruits and roots was sequenced using the Illumina HiSeq X Ten platform (Illumina, San Diego, CA, USA).

### Evaluation of assembly quality

The completeness and accuracy of the genome assembly were evaluated by CEGMA version 2.5 (Parra *et al.*, 2007) and BUSCO version 3.0.2 (Simão *et al.*, 2015). Transcripts from five different tissues and organs were mapped to the assembled genome with GMAP (Wu and Watanabe, 2005) to examine the quality of the genome assembly.

### Annotation of repetitive sequences

A *de novo* repeat database was predicted based on RepeatScout version 1.0.5 (Price *et al.*, 2005), MITE‐Hunter version 1.0 (Han and Wessler, 2010), LTR‐FINDER version 1.0.7 (Xu and Wang, 2007) and PILER version 1.0 (Edgar and Myers, 2005) with default parameters. The predicted repeats from the database were classified using PASTE version 1.0 with default parameters (Hoede *et al.*, 2014). Then, the predicted repeats database combined with Repbase database version 19.06 (Bao *et al.*, 2015) of repetitive DNA elements to a final repeat database. Finally, RepeatMasker program version 4.0.7 (Tarailo‐Graovac and Chen 2009) with the parameters (‐nolow ‐no is ‐norna ‐engine wublast ‐qq –frag 20 000) was used to identify repeat sequences through aligning them against the final repeat database.

### Gene perdition and functional annotations

A combination of *de novo* gene prediction, homology‐based prediction, and RNA‐seq data was used for annotation of protein‐coding genes in the *F*. *nilgerrensis* genome. Genscan version 3.1 (Burge and Karlin, 1997), Augustus version 2.5.5 (Stanke and Waack, 2003), GlimmerHMM version 3.0.4 (Majoros *et al.*, 2004), SNAP version 2006–07‐28 (Johnson *et al.*, 2008) and GeneID version 1.4 (Blanco et al. 2007) were used for the *de novo* gene prediction with default parameters. Sequences of *F. vesca*, *Arabidopsis thaliana*, *Oryza sativa* and *Malus* × *domestica* were used for predicting homologous genes by GeMoMa version 1.3.1 (Keilwagen *et al.*, 2016). The RNA‐seq data were aligned to the *F*. *vesca* reference genome (v1.0) using PASA v2.0.2 (Tang *et al.*, 2015) under default parameters. All predictions by the three methods were combined using EVidenceModeler v1.1.1 (Haas *et al.*, 2008) with default parameters to generate a consensus gene model. PASA pipeline was used for modifying the final gene models.

Gene functions were assigned based on the best match of the alignments against multiple functional databases through BLASTP (Camacho *et al.*, 2009) (E‐value = 1e^‐5^), including the nonredundant protein (Nr) database, TrEMBL, KOG and KEGG (Kanehisa *et al.*, 2016). InterProScan version 4.3 (Quevillon *et al.*, 2005) was used to obtain domain information. Gene sequences were also aligned to the GO database (Consortium 2004) to perform functional annotation via Blast2GO (Conesa *et al.*, 2005). In addition, predicted genes were conducted KOG functional enrichment analysis, KEGG pathway enrichment analysis (Kanehisa *et al.*, 2016), and GO functional enrichment analysis.

### Prediction of noncoding RNA and pseudogenes

miRNAs and rRNAs in the assembled *F*. *nilgerrensis* genome were identified by the INFERNAL software (Nawrocki and Eddy, 2013) against the Rfam 13.0 (Griffiths‐Jones et al. 2005) and miRBase database (Kozomara and Griffiths‐Jones, 2010). The tRNAs were predicted using tRNAscan‐SE version 1.3.1 (Lowe and Eddy, 1997) with the default parameters. The candidate homologous gene sequences were identified by GenBlastA v1.0.4 (She *et al.*, 2009) to mask protein‐coding genes. Then, pseudogenes were predicted by GeneWise v2.4.1 (Birney and Durbin, 2000) with a premature stop premature stop and/or frameshift mutation in the coding region.

### Gene family and phylogenetic analyses

OrthoMCL software version 2.0.9 (Li *et al.*, 2003) was used for identifying gene families/clusters among the protein sequences of 13 genomes, including *F. vesca*, *M.* × *domestica*, *Prunus avium*, *Prunus persica*, *Pyrus Communis*, *Pyrus bretschneideri*, *Rosa chinensis*, *Rubus occidentalis*, *A. thaliana*, *Vitis vinifera*, *Solanum lycopersicum*, *Citrus sinensis and O. sativa*, as well as *F. nilgerrensis*. The parameters of OrthoMCL were as follows: Pep length 10, Stop coden 20, Percent‐MatchCutoff 50, EvalueExponentCutoff −5, Mcl 1.5 #1.2∼4.0. The gene family expansion and contraction analysis was performed using CAFE pipeline v3.1 (De Bie *et al.*, 2006) with the parameters (lambda ‐l 0.002). Phylogenetic tree was constructed using PhyML v3.1 (Guindon et al. 2005) based on 373 single‐copy genes with the parameters (‐gapRatio 0.5 ‐badRatio 0.25 ‐model HKY85 ‐bootstrap 1000). MCMCtree program (Yang, 2007) was used to estimate divergence times with parameters (burn‐in = 10 000, sample‐frequency = 2, sample‐number = 100 000).

### ***Comparative analysis of F*** *.* ***nilgerrensis and F*** *.* ***vesca genome***

The degree of collinearity of *F*. *nilgerrensis* and *F*. *vesca* was detected using MUMmer version 3.23 software (Delcher et al. 2003). Firstly, we used the parameter ‘‐‐mum –p’. Next, we used the ‘delta‐filter ‐i 80 ‐l 20000’ to filter the alignment result. Finally, we used ‘mummerplot ‐‐png ‐p’ to plot the collinearity between *F. nilgerrensis* and *F*. *vesca*. SNPs and indels between *F. nilgerrensis* and *F. vesca* were analysed by MUMmer version 3.23. Firstly, we used the parameter ‘‐maxmatch ‐c 90 ‐l 40’ to align *F. vesca* and *F. nilgerrensis*. Then, we used the ‘delta‐filter −1’ parameter with the one‐to‐one alignment block option to filter the alignment results. We used ‘show‐snp’ to identify SNPs and Indels in the one‐to‐one alignment block (parameter ‐Clr TH). SV between *F*. *nilgerrensis* and *F*. *vesca* was examined by MUMmer version 3.23. We first extracted the alignment blocks of MUMmer version 3.23 with inversions and filtered the blocks with low similarity in the two flanks. The remaining inversion blocks was manually checked and the neighbouring blocks within 50 bp were integrated. Translocation refers to the situation when a DNA fragment occurs in different locations in the two genomes was tested by identifying noncollinear single‐copy homologous blocks (length> 100 bp; identity> 90%) between the *F*. *nilgerrensis* and *F*. *vesca* genomes. The distribution of SV in the exon, promoter and downstream regions of genes in the *F*. *nilgerrensis* genome was detected as described by Delcher *et al. *(2002) and Zhang *et al. *(2016a)

### Quantitative real‐time PCR

Total RNA from different ripening stage fruits (more detailed descriptions were shown in Sample Collection) of *F*. *nilgerrensis* and *F*. *vesca* was extracted using the modified CTAB method as described by Zhang *et al. *(2017). cDNA was synthesized via a PrimeScriptTM RT reagent Kit (TaKaRa, Dalian, China) according to the manufacturer’s protocol. Quantitative real‐time RT‐PCR (qRT‐PCR) was carried out using UltraSYBR Mixture (CWBio, Beijing, China) with gene‐specific primers (Table S10). qRT‐PCR was conducted on the QuantStudioTM6Flex Real‐Time PCR System (Applied Biosystems) in a total volume of 10 μL containing 0.5 μL diluted cDNA, 0.2 μm gene‐specific primers, 3.5 μL ddH_2_O and 5 μL UltraSYBR Mixture. Thermal cycling conditions consisted of a first step of denaturation at 95°C for 10 min, followed by 40 cycles of denaturation for 15 s at 95°C and annealing/extension for 1 min at 60°C. The 2^‐ΔΔCt^ method was used to calculate the relative mRNA levels. All data were normalized first with the level of the 26S internal transcript control and then with the expression of controls. Each sample was examined in triplicate with three biological replicates (Zhang *et al.*, 2016b).

### HPLC analysis

Mature fruits of *F*. *nilgerrensis* and *F*. *vesca* were harvested in February 2019 (more detailed descriptions were shown in Sample Collection). Then, the fruits were ground to a fine powder in liquid nitrogen. 0.2 g powdered sample was soaked in 2 mL methanol solution containing 1% (v/v) hydrochloric acid at 4°C under the dark condition for 48 h. Then, we centrifuged and collected the supernatant. The residues were re‐soaked with 2 mL methanol solution containing 1% hydrochloric acid and then combined the supernatants and added the supernatants to 5 mL.

Anthocyanins were isolated by the Agilent 1100 liquid chromatography equipped with a DAD diode array detector. Pelargonidin‐3‐glucoside and cyanidin‐3‐glucoside were purchased from Shanghai YuanyeBio‐technology Co., Ltd is Shanghai, China. The standards were dissolved in methanol and the concentrations of these standards were 100 μg/mL. The column was DIKMA ODS column (250 mm × 4.6 mm i.d, 5 µm, reverse phase C18 column). The detected wavelength was 520nm and the temperature for column was 25°C. The mobile phase was 1% trifluoroacetic acid water (solvent A) and acetonitrile (solvent B). The elution contained a gradient system over 52 min at a flow rate of 0.7 mL/min. Initial mobile phase composition was 100% B solvent and kept in 45 min, followed by a linear gradient to 45% B solvent in 47 min, and finally 100% B solvent in 52 min. Compositions were identified by comparing their retention times with standard times under the same conditions.

### Dual luciferase reporter system

Promoter region of *FnMYB10* and *FvMYB10* were amplified from the *F*. *nilgerrensis* and *F*. *vesca* genomic DNA, respectively. Both of the fragments were digested with *Xma* I and *Bam*H I, inserted into the pGreenII0800‐LUC vector as reporter plasmids (Hellens *et al.*, 2005). These plasmids were introduced into *Agrobacterium tumefaciens* strain GV3101 by freeze‐thaw method. *Agrobacterium* strain GV3101 carrying the empty vector, plasmids ProFnMYB10‐LUC, and ProFvMYB10‐LUC was cultured to OD_600_ = 1.0 at 28°C, respectively. The cells were harvested and resuspended with medium (1 m MgCl_2_, 100 mm acetosyringone and 1 m MES, pH 5.6). Then, *Agrobacterium* was placed at room temperature without shaking for 2h, and infiltrated into 1‐month‐old *Nicotiana benthamiana* leaves. The infiltrated tobacco was placed in the dark for 24 h and then put them under the light for 48 h. We tested the luciferase signalling by a living fluorescence imager (Lb985, Berthold, Germany). The transcriptional activity of these infiltrated tobacco leaves based on the ratio of luciferase to Renilla (REN) was investigated by Dual Luciferase Reporter Gene assay kit (Beyotime, China). Six biological repeats were measured for each sample. The primers used for the LUC/REN activity analysis were listed in Table S10.

## Conflict of interest

No conflicts of interest are declared.

## Authors’ contributions

Junxiang Zhang and Zhihong Zhang designed the experiments. Yue Ma, Jiahong Wang and Shuang Yu collected plant materials. Junxiang Zhang, Yingying Lei, He Li, Yuexue Liu, Baotian Wang, Hongyan Dai conducted experiments and analysed data. Song Li conducted bioinformatic analysis. Junxiang Zhang, Yan Wang and Zhihong Zhang wrote and modified the manuscript. All authors in this study read and approved the manuscript.

## Supporting information


**Figure S1** *Fragaria nilgerrensis* used in this study which is from Yunnan, China.
**Figure S2** Schematic workflow for the genome assembly of *Fragaria nilgerrensis* from Yunnan, China.
**Figure S3** Mutogram between all chromosomes in the *Fragaria nilgerrensis* genome.
**Figure S4** K‐mer frequency distribution curve (k‐mer=19) of Illumina short reads of the *Fragaria nilgerrensis* genome.
**Figure S5** Gene collinearity between the *Fragaria vesca* and *F. nilgerrensis* genomes.
**Figure S6** Schematic workflow for the gene annotation of the *Fragaria nilgerrensis*.
**Figure S7** Venn diagram of gene annotation of the *Fragaria nilgerrensis* based ab initio gene prediction, homology‐based method and RNA‐seq.
**Figure S8** Gene ontology categories of the annotated genes.
**Figure S9** HPLC elution profile of anthocyanin accumulated in the mature fruit of *Fragaria nilgerrensis* (Fn) and *F. vesca* (Fv).
**Figure S10** The alignment of MYB10 proteins from *Fragaria nilgerrensis* (Fn) and *F. vesca* (Fv) by DNASTAR software.
**Figure S11** The alignment of MYB10 promoter sequences from *Fragaria nilgerrensis* (Fn) and *F. vesca* (Fv) by DNASTAR software.

**Table S1** Genome completeness evaluation of *Fragaria nilgerrensis* based on Illumina sequencing reads.
**Table S2** Genome completeness assessment of *Fragaria nilgerrensis* genome by BUSCO.
**Table S3** Completeness analysis of *Fragaria nilgerrensis* genome based on CEG database.
**Table S4** Gene prediction of *Fragaria nilgerrensis* based on Ab initio, Homology‐based and RNA‐seq method.
**Table S5** Gene functional annotation of *Fragaria nilgerrensis* based on different databases.
**Table S6** Noncoding RNA prediction of *Fragaria nilgerrensis*.
**Table S7** The number and percentage of SNPs types in *Fragaria nilgerrensis* compared with *F. vesca*.
**Table S8** The numbers of SNPs in different chromosome in *Fragaria nilgerrensis* compared with *F. vesca*.
**Table S9** The numbers of Indels in different chromosome in *Fragaria nilgerrensis* compared with *F. vesca*.
**Table S10** Primers used in this study.
**Appendix S1** The sequences of 373 orthologs in 14 species used for analysing the phylogenetic relationships.

**Appendix S1 **The sequences of 373 orthologs in 14 species used for analysing the phylogenetic relationships.

**Appendix S2 **The information of expansion genes in F. nilgerrensis based on KEGG pathway classification in Figure 3a.

**Appendix S3** The information of contraction genes in F. nilgerrensis based on KEGG pathway classification in Figure 3b.

**Appendix S4** The information of unique genes in F*. nilgerrensis* based on KEGG pathway classification in Figure 3c.

## Data Availability

The whole‐genome sequence data reported in this paper have been deposited at DDBJ/ENA/GenBank under accession number WPAB00000000. The version described in this paper is WPAB01000000. The genome assembly and gene annotations have been deposited in the Genome Warehouse in National Genomics Data Center, Beijing Institute of Genomics (BIG), Chinese Academy of Sciences, under accession number GWHABKC00000000 that is publicly accessible at https://bigd.big.ac.cn/gwh.

## References

[pbi13351-bib-0001] Amiri, H. , Davids, W. and Andersson, S.G. (2003) Birth and death of orphan genes in Rickettsia. Mol. Biol. Evol. 20, 1575–1587. 12832625 10.1093/molbev/msg175

[pbi13351-bib-0002] Ban, Y. , Honda, C. , Hatsuyama, Y. , Igarashi, M. , Bessho, H. and Moriguchi, T. (2007) Isolation and functional analysis of a MYB transcription factor gene that is a key regulator for the development of red coloration in apple skin. Plant Cell Physiol. 48, 958–970. 17526919 10.1093/pcp/pcm066

[pbi13351-bib-0003] Bao, W. , Kojima, K.K. and Kohany, O. (2015) Repbase Update, a database of repetitive elements in eukaryotic genomes. Mobile DNA, 6, 11. 26045719 10.1186/s13100-015-0041-9 PMC4455052

[pbi13351-bib-0004] Biemont, C. (2008) Genome size evolution: within‐species variation in genome size. Heredity, 101, 297–298. 18665185 10.1038/hdy.2008.80

[pbi13351-bib-0005] Birney, E. and Durbin, R. (2000) Using GeneWise in the Drosophila annotation experiment. Genome Res. 10, 547–548. 10779496 10.1101/gr.10.4.547 PMC310858

[pbi13351-bib-0006] Blanco, E. , Parra, G. and Guigó, R. (2007) Using geneid to identify genes. Curr. Prot. Bioinform. 18, 4.3.1–4.3.28. 10.1002/0471250953.bi0403s18 18428791

[pbi13351-bib-0007] Bolger, A.M. , Lohse, M. and Usadel, B. (2014) Trimmomatic: a flexible trimmer for Illumina sequence data. Bioinformatics, 30, 2114–2120. 24695404 10.1093/bioinformatics/btu170 PMC4103590

[pbi13351-bib-0008] Bors, B. and Sullivan, J. (1998) Interspecific crossability of nine diploid *Fragaria* species. HortScience, 33, 483b–483.

[pbi13351-bib-0009] Burge, C. and Karlin, S. (1997) Prediction of complete gene structures in human genomic DNA. J. Mol. Biol. 268, 78–94. 9149143 10.1006/jmbi.1997.0951

[pbi13351-bib-0010] Camacho, C. , Coulouris, G. , Avagyan, V. , Ma, N. , Papadopoulos, J. , Bealer, K. and Madden, T.L. (2009) BLAST+: architecture and applications. BMC Bioinformatics, 10, 421. 20003500 10.1186/1471-2105-10-421 PMC2803857

[pbi13351-bib-0011] Casacuberta, E. and Gonzalez, J. (2013) The impact of transposable elements in environmental adaptation. Mol. Ecol. 22, 1503–1517. 23293987 10.1111/mec.12170

[pbi13351-bib-0012] Chagne, D. , Carlisle, C.M. , Blond, C. , Volz, R.K. , Whitworth, C.J. , Oraguzie, N.C. , Crowhurst, R.N. *et al*. (2007) Mapping a candidate gene (*MdMYB10*) for red flesh and foliage colour in apple. BMC Genom. 8, 212. 10.1186/1471-2164-8-212 PMC1939713 17608951

[pbi13351-bib-0013] Chakraborty, M. , Baldwin‐Brown, J.G. , Long, A.D. and Emerson, J.J. (2016) Contiguous and accurate de novo assembly of metazoan genomes with modest long read coverage. Nucleic Acids Res. 44, e147. 27458204 10.1093/nar/gkw654 PMC5100563

[pbi13351-bib-0014] Chambers, A. , Carle, S. , Njuguna, W. , Chamala, S. , Bassil, N. , Whitaker, V.M. , Barbazuk, W.B. and *et al*. (2013) A genome‐enabled, high‐throughput, and multiplexed fingerprinting platform for strawberry (*Fragaria L.*). Mol. Breeding, 31, 615–629.

[pbi13351-bib-0015] Chen, B. , Li, J.F. , Huo, H.Z. , Wan, C.Y. , Zhang, Z. , Qiao, Y.S. and Mi, L. (2015) Estimation of genome size in six wild strawberry species. J. Fruit Sci. 32, 51–56.

[pbi13351-bib-0016] Chuong, E.B. , Elde, N.C. and Feschotte, C. (2017) Regulatory activities of transposable elements: from conflicts to benefits. Nat. Rev. Genet. 18, 71–86. 27867194 10.1038/nrg.2016.139 PMC5498291

[pbi13351-bib-0017] Conesa, A. , Götz, S. , García‐Gómez, J.M. , Terol, J. , Talón, M. and Robles, M. (2005) Blast2GO: a universal tool for annotation, visualization and analysis in functional genomics research. Bioinformatics, 21, 3674–3676. 16081474 10.1093/bioinformatics/bti610

[pbi13351-bib-0018] Daccord, N. , Celton, J.M. , Linsmith, G. , Becker, C. , Choisne, N. , Schijlen, E. , van de Geest, H. *et al*. (2017) High‐quality de novo assembly of the apple genome and methylome dynamics of early fruit development. Nat. Genet. 49, 1099–1106. 28581499 10.1038/ng.3886

[pbi13351-bib-0019] Darrow, G. (1966) The Strawberry. History, Breeding and Physiology. New York: Holt Rinehart and Winston.

[pbi13351-bib-0020] Darwish, O. , Shahan, R. , Liu, Z. , Slovin, J.P. and Alkharouf, N.W. (2015) Re‐annotation of the woodland strawberry (*Fragaria vesca*) genome. BMC Genom. 16, 29. 10.1186/s12864-015-1221-1 PMC4318131 25623424

[pbi13351-bib-0021] De Bie, T. , Cristianini, N. , Demuth, J.P. and Hahn, M.W. (2006) CAFE: a computational tool for the study of gene family evolution. Bioinformatics, 22, 1269–1271. 16543274 10.1093/bioinformatics/btl097

[pbi13351-bib-0022] Delcher, A.L. , Phillippy, A. , Carlton, J. and Salzberg, S.L. (2002) Fast algorithms for large‐scale genome alignment and comparison. Nucleic Acids Res. 30, 2478–2483. 12034836 10.1093/nar/30.11.2478 PMC117189

[pbi13351-bib-0023] Delcher, A.L. , Salzberg, S.L. and Phillippy, A.M. (2003) Using MUMmer to identify similar regions in large sequence sets. Curr. Protoc. Bioinform., 10–3. 10.1002/0471250953.bi1003s00 18428693

[pbi13351-bib-0024] Dong, X. , Wang, Z. , Tian, L. , Zhang, Y. , Qi, D. , Huo, H. , Xu, J. *et al*. (2019) De novo assembly of a wild pear (*Pyrus betuleafolia*) genome. Plant Biotechnol. J. 18, 581–595. 31368610 10.1111/pbi.13226 PMC6953202

[pbi13351-bib-0025] Edgar, R.C. and Myers, E.W. (2005) PILER: identification and classification of genomic repeats. Bioinformatics, 21(suppl_1):152–158. 15961452 10.1093/bioinformatics/bti1003

[pbi13351-bib-0026] Edger, P.P. , VanBuren, R. , Colle, M. , Poorten, T.J. , Wai, C.M. , Niederhuth, C.E. , Alger, E.I. *et al*. (2017) Single‐molecule sequencing and optical mapping yields an improved genome of woodland strawberry (*Fragaria vesca*) with chromosome‐scale contiguity. Gigascience 7, gix124. 10.1093/gigascience/gix124 PMC5801600 29253147

[pbi13351-bib-0027] Edger, P.P. , Poorten, T.J. , VanBuren, R. , Hardigan, M.A. , Colle, M. , McKain, M.R. , Smith, R.D. *et al*. (2019) Origin and evolution of the octoploid strawberry genome. Nat. Genet. 51, 541–547. 30804557 10.1038/s41588-019-0356-4 PMC6882729

[pbi13351-bib-0028] El‐Sharkawy, I. , Liang, D. and Xu, K. (2015) Transcriptome analysis of an apple (*Malus*×*domestica*) yellow fruit somatic mutation identifies a gene network module highly associated with anthocyanin and epigenetic regulation. J. Exp. Bot. 66, 7359–7376. 26417021 10.1093/jxb/erv433 PMC4765799

[pbi13351-bib-0029] English, A.C. , Richards, S. , Han, Y. , Wang, M. , Vee, V. , Qu, J. , Qin, X. *et al*. (2012) Mind the gap: upgrading genomes with Pacific Biosciences RS long‐read sequencing technology. PLoS ONE, 7, e47768. 23185243 10.1371/journal.pone.0047768 PMC3504050

[pbi13351-bib-0030] Espley, R.V. , Hellens, R.P. , Putterill, J. , Stevenson, D.E. , Kutty‐Amma, S. and Allan, A.C. (2007) Red colouration in apple fruit is due to the activity of the MYB transcription factor, MdMYB10. Plant J. 49, 414–427. 17181777 10.1111/j.1365-313X.2006.02964.x PMC1865000

[pbi13351-bib-0031] Espley, R.V. , Brendolise, C. , Chagné, D. , Kutty‐Amma, S. , Green, S. , Volz, R. , Putterill, J. *et al*. (2009) Multiple repeats of a promoter segment causes transcription factor autoregulation in red apples. Plant Cell, 21, 168–183. 19151225 10.1105/tpc.108.059329 PMC2648084

[pbi13351-bib-0032] Folta, K.M. and Davis, T.M. (2006) Strawberry genes and genomics. Crit. Rev. Plant Sci. 25, 399–415.

[pbi13351-bib-0033] Gao, F. , Wang, X. , Li, X. , Xu, M. , Li, H. , Abla, M. , Sun, H. *et al*. (2018) Long‐read sequencing and de novo genome assembly of *Ammopiptanthus nanus*, a desert shrub. GigaScience, 7, giy074. 29917074 10.1093/gigascience/giy074 PMC6048559

[pbi13351-bib-0034] Gene Ontology Consortium (2004) The Gene Ontology (GO) database and informatics resource. Nucleic Acids Res. 32, 258–261. 10.1093/nar/gkh036 PMC308770 14681407

[pbi13351-bib-0035] Griffiths‐Jones, S. , Moxon, S. , Marshall, M. , Khanna, A. , Eddy, S.R. , and Bateman , A. (2005) Rfam: annotating non‐coding RNAs in complete genomes. Nucleic Acids Res. 33(suppl_1), 121–124. 10.1093/nar/gki081 PMC540035 15608160

[pbi13351-bib-0036] Guindon, S. , Lethiec, F. , Duroux, P. and Gascuel, O. (2005) PHYML Online‐‐a web server for fast maximum likelihood‐based phylogenetic inference. Nucleic Acids Res. 33, 557–559. 10.1093/nar/gki352 PMC1160113 15980534

[pbi13351-bib-0037] Guo, R. , Xue, L. , Luo, G. , Zhang, T. and Lei, J. (2017) Investigation and taxonomy of wild Fragaria resources in Tibet, China. Gen. Resour. Crop Evol. 65, 405–415.

[pbi13351-bib-0038] Haas, B.J. , Salzberg, S.L. , Zhu, W. , Pertea, M. , Allen, J.E. , Orvis, J. , White, O. *et al*. (2008) Automated eukaryotic gene structure annotation using EVidenceModeler and the Program to Assemble Spliced Alignments. Genome Biol. 9, R7. 18190707 10.1186/gb-2008-9-1-r7 PMC2395244

[pbi13351-bib-0039] Han, Y. and Wessler, S.R. (2010) MITE‐Hunter: a program for discovering miniature inverted‐repeat transposable elements from genomic sequences. Nucleic Acids Res. 38, 199–199. 10.1093/nar/gkq862 PMC3001096 20880995

[pbi13351-bib-0040] Hancock, J. (1999) Strawberries. Cambridge, MA: CABI Pub University Press, 237.

[pbi13351-bib-0041] Hancock, J. and Luby, J. (1993) Genetic Resources at Our Doorstep: the wild strawberries. Bioscience, 43, 141–147.

[pbi13351-bib-0042] Hariharan, R. and Toyama, K. (2004) Project Lachesis: parsing and modeling location histories. International Conference on Geographic Information Science, Springer, Berlin, Heidelberg: 106–124.

[pbi13351-bib-0043] Harrison, R.E. and Luby, J.J. (1997) Chloroplast DNA restriction fragment variation among strawberry (*Fragaria* spp.) taxa. J. Am. Soc. Horticult. Sci. 122, 63–68.

[pbi13351-bib-0044] Hellens, R.P. , Allan, A.C. , Friel, E.N. , Bolitho, K. , Grafton, K. , Templeton, M.D. , Karunairetnam, S. *et al*. (2005) Transient expression vectors for functional genomics, quantification of promoter activity and RNA silencing in plants. Plant Methods, 1, 13. 16359558 10.1186/1746-4811-1-13 PMC1334188

[pbi13351-bib-0045] Higo, K. , Ugawa, Y. , Iwamoto, M. and Korenaga, T. (1999) Plant cis‐acting regulatory DNA elements (PLACE) database: 1999. Nucleic Acids Res. 27, 297–300. 9847208 10.1093/nar/27.1.297 PMC148163

[pbi13351-bib-0046] Hirakawa, H. , Shirasawa, K. , Kosugi, S. , Tashiro, K. , Nakayama, S. , Yamada, M. , Kohara, M. *et al*. (2014) Dissection of the octoploid strawberry genome by deep sequencing of the genomes of *Fragaria* species. DNA Res. 21, 169–181. 24282021 10.1093/dnares/dst049 PMC3989489

[pbi13351-bib-0047] Hoede, C. , Arnoux, S. , Moisset, M. , Chaumier, T. , Inizan, O. , Jamilloux, V. and Quesneville, H. (2014) PASTEC: an automatic transposable element classification tool. PLoS ONE, 9, e91929. 24786468 10.1371/journal.pone.0091929 PMC4008368

[pbi13351-bib-0048] Huang, L. , Feng, G. , Yan, H. , Zhang, Z. , Bushman, B.S. , Wang, J. , Bombarely, A. *et al*. (2019) Genome assembly provides insights into the genome evolution and flowering regulation of orchardgrass. Plant Biotechnol. J. 18, 373–388. 31276273 10.1111/pbi.13205 PMC6953241

[pbi13351-bib-0049] Hummer, K.E. and Hancock, J. (2009) Strawberry Genomics: Botanical History, Cultivation, Traditional Breeding, and New Technologies. Genetics and Genomics of Rosaceae, pp. 413–435. New York, NY: Springer.

[pbi13351-bib-0050] Jia, H. , Wang, Y. , Sun, M. , Li, B. , Han, Y. , Zhao, Y. , Li, X. *et al*. (2013) Sucrose functions as a signal involved in the regulation of strawberry fruit development and ripening. New Phytol. 198, 453–465. 23425297 10.1111/nph.12176

[pbi13351-bib-0051] Johnson, A.D. , Handsaker, R.E. , Pulit, S.L. , Nizzari, M.M. , O'Donnell, C.J. and De Bakker, P.I. (2008) SNAP: a web‐based tool for identification and annotation of proxy SNPs using HapMap. Bioinformatics, 24, 2938–2939. 18974171 10.1093/bioinformatics/btn564 PMC2720775

[pbi13351-bib-0052] Kadomura‐Ishikawa, Y. , Miyawaki, K. , Takahashi, A. , Masuda, T. and Noji, S. (2014) Light and abscisic acid independently regulated *FaMYB10* in *Fragaria× ananassa* fruit. Planta, 241, 953–965. 25534946 10.1007/s00425-014-2228-6

[pbi13351-bib-0053] Kanehisa, M. , Sato, Y. , Kawashima, M. , Furumichi, M. and Tanabe, M. (2016) KEGG as a reference resource for gene and protein annotation. Nucleic Acids Res. 44, 457–462. 10.1093/nar/gkv1070 PMC4702792 26476454

[pbi13351-bib-0054] Keilwagen, J. , Wenk, M. , Erickson, J.L. , Schattat, M.H. , Grau, J. and Hartung, F. (2016) Using intron position conservation for homology‐based gene prediction. Nucleic Acids Res. 44, 89–89. 10.1093/nar/gkw092 PMC4872089 26893356

[pbi13351-bib-0055] Koren, S. , Walenz, B.P. , Berlin, K. , Miller, J.R. , Bergman, N.H. and Phillippy, A.M. (2017) Canu: scalable and accurate long‐read assembly via adaptive k‐mer weighting and repeat separation. Genome Res. 27, 722–736. 28298431 10.1101/gr.215087.116 PMC5411767

[pbi13351-bib-0056] Kozomara, A. and Griffiths‐Jones, S. (2010) miRBase: integrating microRNA annotation and deep‐sequencing data. Nucleic Acids Res. 39, (suppl_1):152–157. 10.1093/nar/gkq1027 PMC3013655 21037258

[pbi13351-bib-0057] Lei, J.J. , Xue, L. , Guo, R.X. and Dai, H.P. (2017) The *Fragaria* species native to China and their geographical distribution. Acta Hort. 1156, 37–46.

[pbi13351-bib-0058] Li, H. (2013) Aligning sequence reads, clone sequences and assembly contigs with BWA‐MEM. arXiv:1303.3997.

[pbi13351-bib-0059] Li, L. , Stoeckert, C.J. and Roos, D.S. (2003) OrthoMCL: identification of ortholog groups for eukaryotic genomes. Genome Res. 13, 2178–2189. 12952885 10.1101/gr.1224503 PMC403725

[pbi13351-bib-0060] Li, Y. , Wei, W. , Feng, J. , Luo, H. , Pi, M. , Liu, Z. and Kang, C. (2017) Genome re‐annotation of the wild strawberry *Fragaria vesca* using extensive Illumina‐ and SMRT‐based RNA‐seq datasets. DNA Res. 25, 61–70. 10.1093/dnares/dsx038 PMC5824900 29036429

[pbi13351-bib-0061] Li, Z.W. , Hou, X.H. , Chen, J.F. , Xu, Y.C. , Wu, Q. , Gonzalez, J. and Guo, Y.L. (2018) Transposable elements contribute to the adaptation of *Arabidopsis thaliana* . Genome Biol. Evol. 10, 2140–2150. 30102348 10.1093/gbe/evy171 PMC6117151

[pbi13351-bib-0062] Lin‐Wang, K. , McGhie, T.K. , Wang, M. , Liu, Y. , Warren, B. , Storey, R. , Espley, R.V. *et al*. (2014) Engineering the anthocyanin regulatory complex of strawberry *(Fragaria vesca*). Front. Plant Sci. 5, 651. 25477896 10.3389/fpls.2014.00651 PMC4237049

[pbi13351-bib-0063] Liston, A. , Cronn, R. and Ashman, T.L. (2014) Fragaria: a genus with deep historical roots and ripe for evolutionary and ecological insights. Am. J. Bot. 101, 1686–1699. 25326614 10.3732/ajb.1400140

[pbi13351-bib-0064] Liu, B. , Shi, Y. , Yuan, J. , Hu, X. , Zhang, H. , Li, N. , Li, Z. *et al*. (2013) Estimation of genomic characteristics by analyzing k‐mer frequency in de novo genome projects. arXiv:1308.2012.

[pbi13351-bib-0065] Liu, B. , Poulsen, E.G. and Davis, T.M. (2016) Insight into octoploid strawberry (*Fragaria*) subgenome composition revealed by GISH analysis of pentaploid hybrids. Genome, 59, 79–86. 26835888 10.1139/gen-2015-0116

[pbi13351-bib-0066] Liu, M. , Li, Y. , Ma, Y. , Zhao, Q. , Stiller, J. , Feng, Q. , Tian, Q. *et al*. (2019) The draft genome of a wild barley genotype reveals its enrichment in genes related to biotic and abiotic stresses compared to cultivated barley. Plant Biotechnol. J. 18, 443–456. 31314154 10.1111/pbi.13210 PMC6953193

[pbi13351-bib-0067] Lowe, T.M. and Eddy, S.R. (1997) tRNAscan‐SE: a program for improved detection of transfer RNA genes in genomic sequence. Nucleic Acids Res. 25, 955–964. 9023104 10.1093/nar/25.5.955 PMC146525

[pbi13351-bib-0068] Luo, G. , Xue, L. , Guo, R. , Ding, Y. , Xu, W. and Lei, J. (2018) Creating interspecific hybrids with improved cold resistance in *Fragaria* . Sci. Hortic. 234, 1–9.

[pbi13351-bib-0069] Luo, Y. , Lin, Y. , Mo, F. , Ge, C. , Jiang, L. , Zhang, Y. , Chen, Q. *et al*. (2019) Sucrose promotes strawberry fruit ripening and affects ripening‐related processes. Int. J. Genom. 2019, 1–14. 10.1155/2019/9203057 PMC6886322 31828083

[pbi13351-bib-0070] Majoros, W.H. , Pertea, M. and Salzberg, S.L. (2004) TigrScan and GlimmerHMM: two open source *ab initio* eukaryotic gene‐finders. Bioinformatics,20, 2878–2879. 15145805 10.1093/bioinformatics/bth315

[pbi13351-bib-0071] Martin, M. (2011) Cutadapt removes adapter sequences from high‐throughput sequencing reads. EMBnet. J. 17, 10–12.

[pbi13351-bib-0072] Medina‐Puche, L. , Cumplido‐Laso, G. , Amil‐Ruiz, F. , Hoffmann, T. , Ring, L. , Rodríguez‐Franco, A. , Caballero, J.L. *et al*. (2015) MYB10 plays a major role in the regulation of flavonoid/phenylpropanoid metabolism during ripening of *Fragaria× ananassa* fruits. J. Exp. Bot. 65, 401–417. 10.1093/jxb/ert377 24277278

[pbi13351-bib-0073] Michael, T.P. and VanBuren, R. (2015) Progress, challenges and the future of crop genomes. Curr. Opin. Plant Biol. 24, 71–81. 25703261 10.1016/j.pbi.2015.02.002

[pbi13351-bib-0074] Michael, T.P. , Jupe, F. , Bemm, F. , Motley, S.T. , Sandoval, J.P. , Lanz, C. , Loudet, O. *et al*. (2018) High contiguity Arabidopsis thaliana genome assembly with a single nanopore flow cell. Nat. Commun. 9, 541. 29416032 10.1038/s41467-018-03016-2 PMC5803254

[pbi13351-bib-0075] Nathewet, P. , Yanagi, T. , Hummer, K.E. and Iwatsubo, S.K. (2009) Karyotype analysis in wild diploid, tetraploid and hexaploid strawberries, *Fragaria* (*Rosaceae*). Cytologia, 74, 355–364.

[pbi13351-bib-0076] Nawrocki, E.P. and Eddy, S.R. (2013) Infernal 1.1: 100‐fold faster RNA homology searches. Bioinformatics, 29, 2933–2935. 24008419 10.1093/bioinformatics/btt509 PMC3810854

[pbi13351-bib-0077] Niu, X.M. , Xu, Y.C. , Li, Z.W. , Bian, Y.T. , Hou, X.H. , Chen, J.F. , Zou, Y.P. *et al*. (2019) Transposable elements drive rapid phenotypic variation in *Capsella rubell*a. Proc. Natl Acad. Sci. USA, 116, 6908–6913. 30877258 10.1073/pnas.1811498116 PMC6452725

[pbi13351-bib-0078] Njuguna, W. , Liston, A. , Cronn, R. , Ashman, T.L. and Bassil, N. (2013) Insights into phylogeny, sex function and age of *Fragaria* based on whole chloroplast genome sequencing. Mol. Phylogenet. Evol. 66, 17–29. 22982444 10.1016/j.ympev.2012.08.026

[pbi13351-bib-0079] Noguchi, Y. , Mochizuki, T. and Sone, K. (2002) Breeding of a new aromatic strawberry by interspecific hybridization *Fragaria × ananassa* × *F. nilgerrensis* . J. Japanese Soc. Horticult. Sci., 71, 208–213.

[pbi13351-bib-0080] Parra, G. , Bradnam, K. and Korf, I. (2007) CEGMA: a pipeline to accurately annotate core genes in eukaryotic genomes. Bioinformatics, 23, 1061–1067. 17332020 10.1093/bioinformatics/btm071

[pbi13351-bib-0081] Potter, D. , Luby, J.J. and Harrison, R.E. (2000) Phylogenetic relationships among species of *Fragaria* (*Rosaceae*) inferred from non‐coding nuclear and chloroplast DNA sequences. Syst. Bot. 25, 337–349.

[pbi13351-bib-0082] Price, A.L. , Jones, N.C. and Pevzner, P.A. (2005) De novo identification of repeat families in large genomes. Bioinformatics 21, (suppl_1), 351–358. 10.1093/bioinformatics/bti1018 15961478

[pbi13351-bib-0083] Qiao, Q. , Xue, L. , Wang, Q. , Sun, H. , Zhong, Y. , Huang, J. , Lei, J. *et al*. (2016) Comparative transcriptomics of strawberries (*Fragaria* spp.) provides insights into evolutionary patterns. Front. Plant Sci. 7, 1839. 28018379 10.3389/fpls.2016.01839 PMC5156730

[pbi13351-bib-0084] Quevillon, E. , Silventoinen, V. , Pillai, S. , Harte, N. , Mulder, N. , Apweiler, R. and Lopez, R. (2005) InterProScan: protein domains identifier. Nucleic Acids Res. 33, (suppl_2), 116–120. 10.1093/nar/gki442 PMC1160203 15980438

[pbi13351-bib-0085] Rho, I.R. , Hwang, Y.J. , Lee, H.I. , Lee, C.‐H. and Lim, K.B. (2012) Karyotype analysis using FISH (fluorescence in situ hybridization) in *Fragaria* . Sci. Hortic. 136, 95–100.

[pbi13351-bib-0086] Rousseau‐Gueutin, M. , Gaston, A. , Ainouche, A. , Ainouche, M.L. , Olbricht, K. , Staudt, G. , Richard, L. *et al*. (2009) Tracking the evolutionary history of polyploidy in *Fragaria L.* (strawberry): new insights from phylogenetic analyses of low‐copy nuclear genes. Mol. Phylogenet. Evol. 51, 515–530. 19166953 10.1016/j.ympev.2008.12.024

[pbi13351-bib-0087] Saint‐Oyant, L.H. , Ruttink, T. , Hamama, L. , Kirov, I. , Lakhwani, D. , Zhou, N.N. , Bourke, P.M. *et al*. (2018) A high‐quality genome sequence of *Rosa chinensis* to elucidate ornamental traits. Nat. Plants, 4, 473–484. 29892093 10.1038/s41477-018-0166-1 PMC6786968

[pbi13351-bib-0088] Sangiacomo, M. and Sullivan, J. (1994) Introgression of wild species into the cultivated strawberry using synthetic octoploids. Theor. Appl. Genet. 88, 349–354. 24186018 10.1007/BF00223644

[pbi13351-bib-0089] Sargent, D.J. , Geibel, M. , Hawkins, J.A. , Wilkinson, M.J. , Battey, N.H. and Simpson, D.W. (2004) Quantitative and qualitative differences in morphological traits revealed between diploid *Fragaria* species. Ann. Bot. 94, 787–796. 15469944 10.1093/aob/mch217 PMC4242284

[pbi13351-bib-0090] Schulz, P. , Herde, M. and Romeis, T. (2013) Calcium‐dependent protein kinases: hubs in plant stress signaling and development. Plant Physiol. 163, 523–530. 24014579 10.1104/pp.113.222539 PMC3793034

[pbi13351-bib-0091] Servant, N. , Varoquaux, N. , Lajoie, B.R. , Viara, E. , Chen, C.‐J. , Vert, J.‐P. , Heard, E. *et al*. (2015) HiC‐Pro: an optimized and flexible pipeline for Hi‐C data processing. Genome Biol. 16, 259. 26619908 10.1186/s13059-015-0831-x PMC4665391

[pbi13351-bib-0092] She, R. , Chu, J.S.‐C. , Wang, K. , Pei, J. and Chen, N. (2009) GenBlastA: enabling BLAST to identify homologous gene sequences. Genome Res. 19, 143–149. 18838612 10.1101/gr.082081.108 PMC2612959

[pbi13351-bib-0093] Shulaev, V. , Sargent, D.J. , Crowhurst, R.N. , Mockler, T.C. , Folkerts, O. , Delcher, A.L. , Jaiswal, P. *et al*. (2011) The genome of woodland strawberry (*Fragaria vesca*). Nat. Genet. 43, 109–116. 21186353 10.1038/ng.740 PMC3326587

[pbi13351-bib-0094] Simão, F.A. , Waterhouse, R.M. , Ioannidis, P. , Kriventseva, E.V. and Zdobnov, E.M. (2015) BUSCO: assessing genome assembly and annotation completeness with single‐copy orthologs. Bioinformatics, 31, 3210–3212. 26059717 10.1093/bioinformatics/btv351

[pbi13351-bib-0095] Song, S. , Tian, D. , Zhang, Z. , Hu, S. and Yu, J. (2018) Rice genomics: over the past two decades and into the future. Genom. Proteom. Bioinform. 16, 397–404. 10.1016/j.gpb.2019.01.001 PMC6411948 30771506

[pbi13351-bib-0096] Stanke, M. and Waack, S. (2003) Gene prediction with a hidden Markov model and a new intron submodel. Bioinformatics, 19(Suppl 2), 215–225. 10.1093/bioinformatics/btg1080 14534192

[pbi13351-bib-0097] Staudt, G. (1989) The species of Fragaria, their taxonomy and geographical distribution. Acta Hort. 265, 23–34.

[pbi13351-bib-0098] Staudt, G. (1999) Systematics and Geographic Distribution of the American Strawberry Species. Berkeley, CA: University of California Publications in Botany.

[pbi13351-bib-0099] Staudt, G. (2009) Strawberry biogeography, genetics and systematics. Acta Hort. 842, 71–84.

[pbi13351-bib-0100] Sun, C. , Palmqvist, S. , Olsson, H. , Boren, M. , Ahlandsberg, S. and Jansson, C. (2003) A novel WRKY transcription factor, SUSIBA2, participates in sugar signaling in barley by binding to the sugar‐responsive elements of the iso1 promoter. Plant Cell, 15, 2076–2092. 12953112 10.1105/tpc.014597 PMC181332

[pbi13351-bib-0101] Tang, S. , Lomsadze, A. and Borodovsky, M. (2015) Identification of protein coding regions in RNA transcripts. Nucleic Acids Res. 43, e78. 25870408 10.1093/nar/gkv227 PMC4499116

[pbi13351-bib-0102] Tarailo‐Graovac, M. and Chen, N. (2009) Using RepeatMasker to identify repetitive elements in genomic sequences. Curr. Protoc. Bioinform. 25, 4–10. 10.1002/0471250953.bi0410s25 19274634

[pbi13351-bib-0103] Teh, B.T. , Lim, K. , Yong, C.H. , Ng, C.C.Y. , Rao, S.R. , Rajasegaran, V. , Lim, W.K. *et al*. (2017) The draft genome of tropical fruit durian (*Durio zibethinus*). Nat. Genet. 49, 1633–1641. 28991254 10.1038/ng.3972

[pbi13351-bib-0104] Tenaillon, M.I. , Hufford, M.B. , Gaut, B.S. and Ross‐Ibarra, J. (2011) Genome size and transposable element content as determined by high‐throughput sequencing in maize and Zea luxurians. Genome Biol. Evol. 3, 219–229. 21296765 10.1093/gbe/evr008 PMC3068001

[pbi13351-bib-0105] Terzaghi, W.B. and Cashmore, A.R. (1995) Light‐regulated transcription. Annu. Rev. Plant Biol. 46, 445–474.

[pbi13351-bib-0106] Walker, B.J. , Abeel, T. , Shea, T. , Priest, M. , Abouelliel, A. , Sakthikumar, S. , Cuomo, C.A. *et al*. (2014) Pilon: an integrated tool for comprehensive microbial variant detection and genome assembly improvement. PLoS ONE, 9, e112963. 25409509 10.1371/journal.pone.0112963 PMC4237348

[pbi13351-bib-0107] Wang, Z. , Meng, D. , Wang, A. , Li, T. , Jiang, S. , Cong, P. and Li, T. (2013) The methylation of the *PcMYB10* promoter is associated with green‐skinned sport in Max Red Bartlett pear. Plant Physiol. 162, 885–896. 23629835 10.1104/pp.113.214700 PMC3668077

[pbi13351-bib-0108] Wang, J. , Yu, H.M. , Cai, W.J. and Zhao, M.Z. (2017) Studies on diversity of wild *Fragaria* species in Yunnan, China. Acta Hort., 1156, 103–110.

[pbi13351-bib-0109] Wang, H. , Zhang, H. , Yang, Y. , Li, M. , Zhang, Y. , Liu, J. , Dong, J. *et al*. (2019) The control of red colour by a family of MYB transcription factors in octoploid strawberry (*Fragaria* × *ananassa*) fruits. Plant Biotechnol. J. 10.1111/pbi.13282 PMC7152614 31647169

[pbi13351-bib-0110] Wu, T.D. and Watanabe, C.K. (2005) GMAP: a genomic mapping and alignment program for mRNA and EST sequences. Bioinformatics, 21, 1859–1875. 15728110 10.1093/bioinformatics/bti310

[pbi13351-bib-0111] Xu, Z. and Wang, H. (2007) LTR_FINDER: an efficient tool for the prediction of full‐length LTR retrotransposons. Nucleic Acids Res. 35, (suppl_2), 265–268. 10.1093/nar/gkm286 PMC1933203 17485477

[pbi13351-bib-0112] Xu, Y. , Wu, G. , Hao, B. , Chen, L. , Deng, X. and Xu, Q. (2015) Identification, characterization and expression analysis of lineage‐specific genes within sweet orange (*Citrus sinensis*). BMC Genom. 16, 995. 10.1186/s12864-015-2211-z PMC4657247 26597278

[pbi13351-bib-0113] Yang, Z. (2007) PAML 4: phylogenetic analysis by maximum likelihood. Mol. Biol. Evol. 24, 1586–1591. 17483113 10.1093/molbev/msm088

[pbi13351-bib-0114] Yarnell, S. (1928) Notes on the somatic chromosomes of the seven‐chromosome group of *Fragaria* . Genetic 14, 78–84. 10.1093/genetics/14.1.78 PMC1201025 17246569

[pbi13351-bib-0115] Zhai, R. , Wang, Z. , Zhang, S. , Meng, G. , Song, L. , Wang, Z. , Li, P. *et al*. (2015) Two MYB transcription factors regulate flavonoid biosynthesis in pear fruit (*Pyrus bretschneideri* Rehd.). J. Exp. Bot. 67, 1275–1284. 26687179 10.1093/jxb/erv524

[pbi13351-bib-0116] Zhang, J. , Chen, L.L. , Xing, F. , Kudrna, D.A. , Yao, W. , Copetti, D. , Mu, T. *et al*. (2016a) Extensive sequence divergence between the reference genomes of two elite indica rice varieties Zhenshan 97 and Minghui 63. Proc. Natl Acad. Sci. USA, 113, 5163–5171. 10.1073/pnas.1611012113 PMC5024649 27535938

[pbi13351-bib-0117] Zhang, J. , Yuan, H. , Yang, Y. , Fish, T. , Lyi, S.M. , Thannhauser, T.W. , Zhang, L. *et al*. (2016b) Plastid ribosomal protein S5 is involved in photosynthesis, plant development, and cold stress tolerance in Arabidopsis. J. Exp. Bot. 67, 2731–2744. 27006483 10.1093/jxb/erw106 PMC4861020

[pbi13351-bib-0118] Zhang, J. , Zhang, Y. , Dou, Y. , Li, W. , Wang, S. , Shi, W. , Sun, Y. *et al*. (2017) Single nucleotide mutation in *FvMYB10* may lead to the yellow fruit in *Fragaria vesca* . Mol. Breeding, 37, 35.

[pbi13351-bib-0119] Zhang, L. , Hu, J. , Han, X. , Li, J. , Gao, Y. , Richards, C.M. , Zhang, C. *et al*. (2019) A high‐quality apple genome assembly reveals the association of a retrotransposon and red fruit colour. Nat. Commun. 10, 1494. 30940818 10.1038/s41467-019-09518-x PMC6445120

