# Supplementary material for: The high‐quality genome of diploid strawberry (Fragaria nilgerrensis) provides new insights into anthocyanin accumulation
Source: Plant Biotechnol J. 2020 Feb 15;18(9):1908–24. doi: 10.1111/pbi.13351 (PMC7415782; doi:10.1111/pbi.13351)
Supplement: Supplementary file 1 — Figure S1 Fragaria nilgerrensis used in this study which is from Yunnan, China. Figure S2 Schematic workflow for the genome assembly of Fragaria nilgerrensis from Yunnan, China. Figure S3 Mutogram between all chromosomes in the Fragaria nilgerrensis genome. Figure S4 K‐mer frequency distribution curve (k‐mer=19) of Illumina short reads of the Fragaria nilgerrensis genome. Figure S5 Gene collinearity between the Fragaria vesca and F. nilgerrensis genomes. Figure S6 Schematic workflow for the gene annotation of the Fragaria nilgerrensis. Figure S7 Venn diagram of gene annotation of the Fragaria nilgerrensis based ab initio gene prediction, homology‐based method and RNA‐seq. Figure S8 Gene ontology categories of the annotated genes. Figure S9 HPLC elution profile of anthocyanin accumulated in the mature fruit of Fragaria nilgerrensis (Fn) and F. vesca (Fv). Figure S10 The alignment of MYB10 proteins from Fragaria nilgerrensis (Fn) and F. vesca (Fv) by DNASTAR software. Figure S11 The alignment of MYB10 promoter sequences from Fragaria nilgerrensis (Fn) and F. vesca (Fv) by DNASTAR software. [file PBI-18-1908-s004.docx]

**
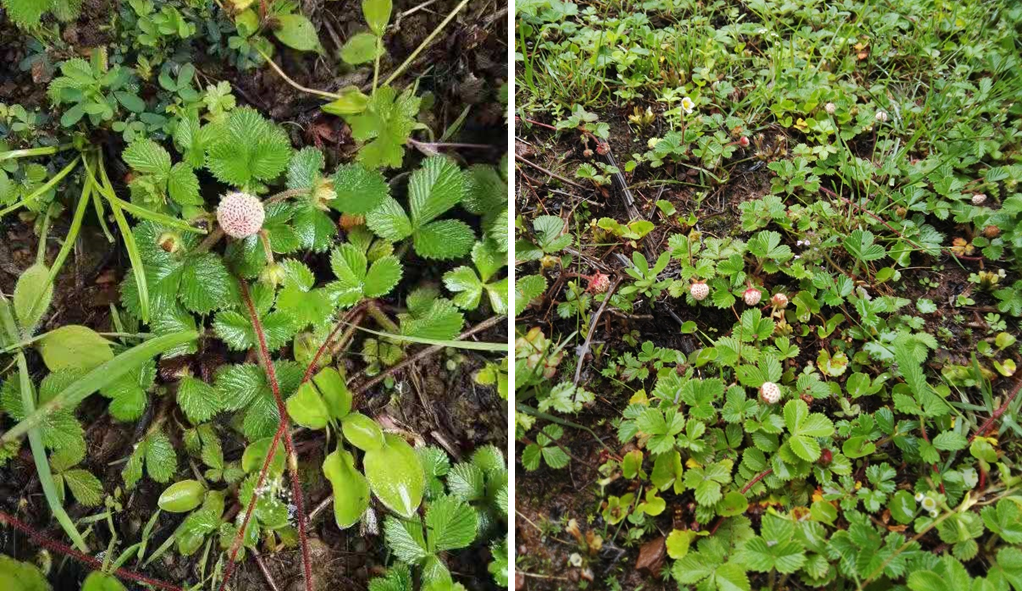
**

**Figure S1.** *Fragaria nilgerrensis* used in this study which is from Yunnan, China.

**Falcon and CANU Assembly**

**Pacbio Long Reads**

**De novo Contigs Assembly**

**Quickmerge**

**llumina Short Reads**

**PILON**

**K-mer analysis**

**BUSCO**

**Error Correction**

**Genome size, Repeats ratio, Heterozygosity ratio, and GC contents**

**Genome**

**Chromosome**s

**PBjelly Gap filling**

**HiC-Pro**

**LACHESIS**

**Scaffolds**

**CEGMA, BUSCO, Ilumina Short Reads Evaluation**

**Contigs**

**Hi-C library Construction and Sequencing**

**Figure S2.** Schematic workflow for the genome assembly of *Fragaria nilgerrensis* from Yunnan, China.


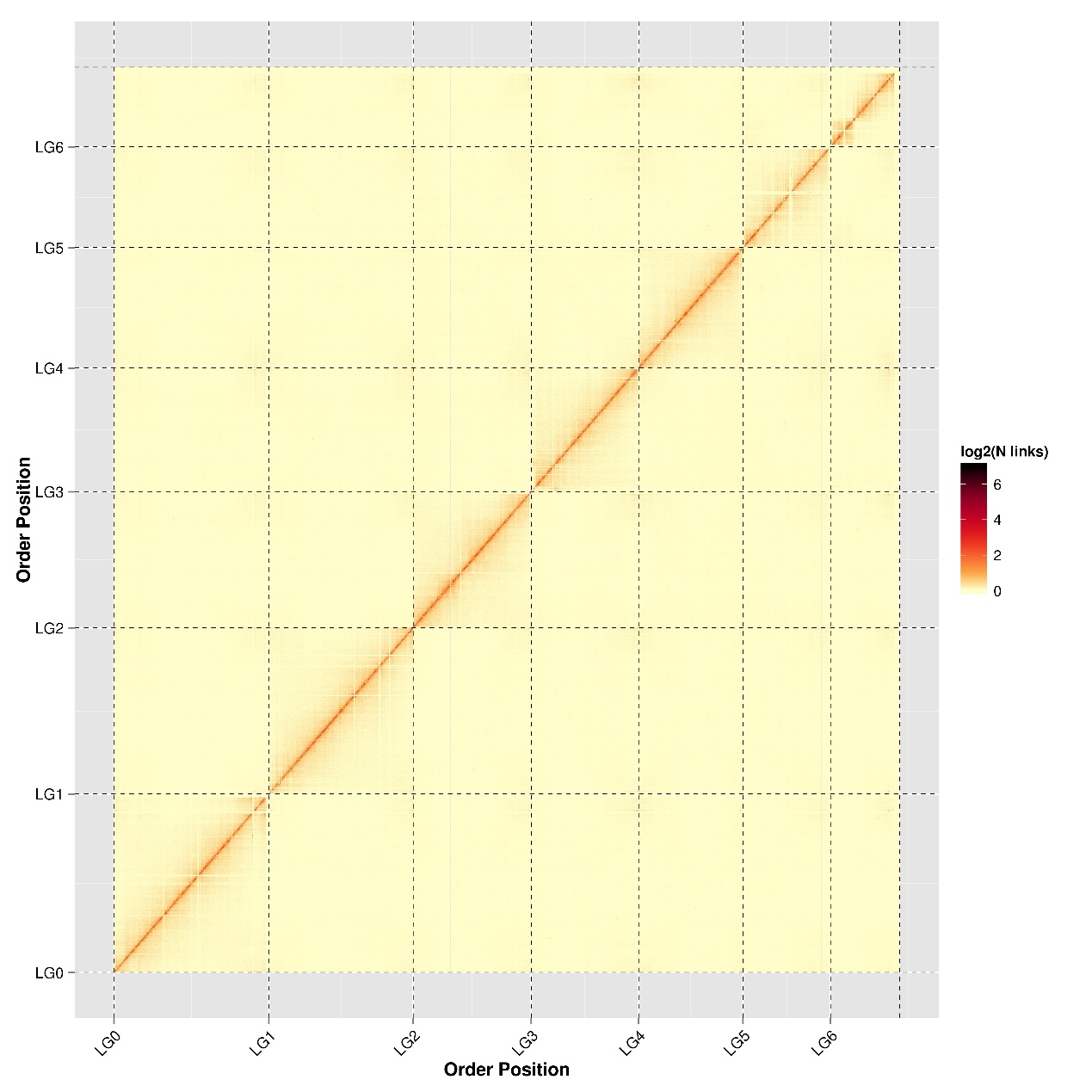


**Figure S3.** Mutogram between all chromosomes in the *Fragaria nilgerrensis* genome. To test the accuracy of the Hi-C assembly, the interaction between chromosomes was drawn based on the frequency of interaction between chromosomes. The deeper the colour, the higher the frequency of interaction. The frequency of interaction within chromosomes is larger than the interaction between chromosomes.


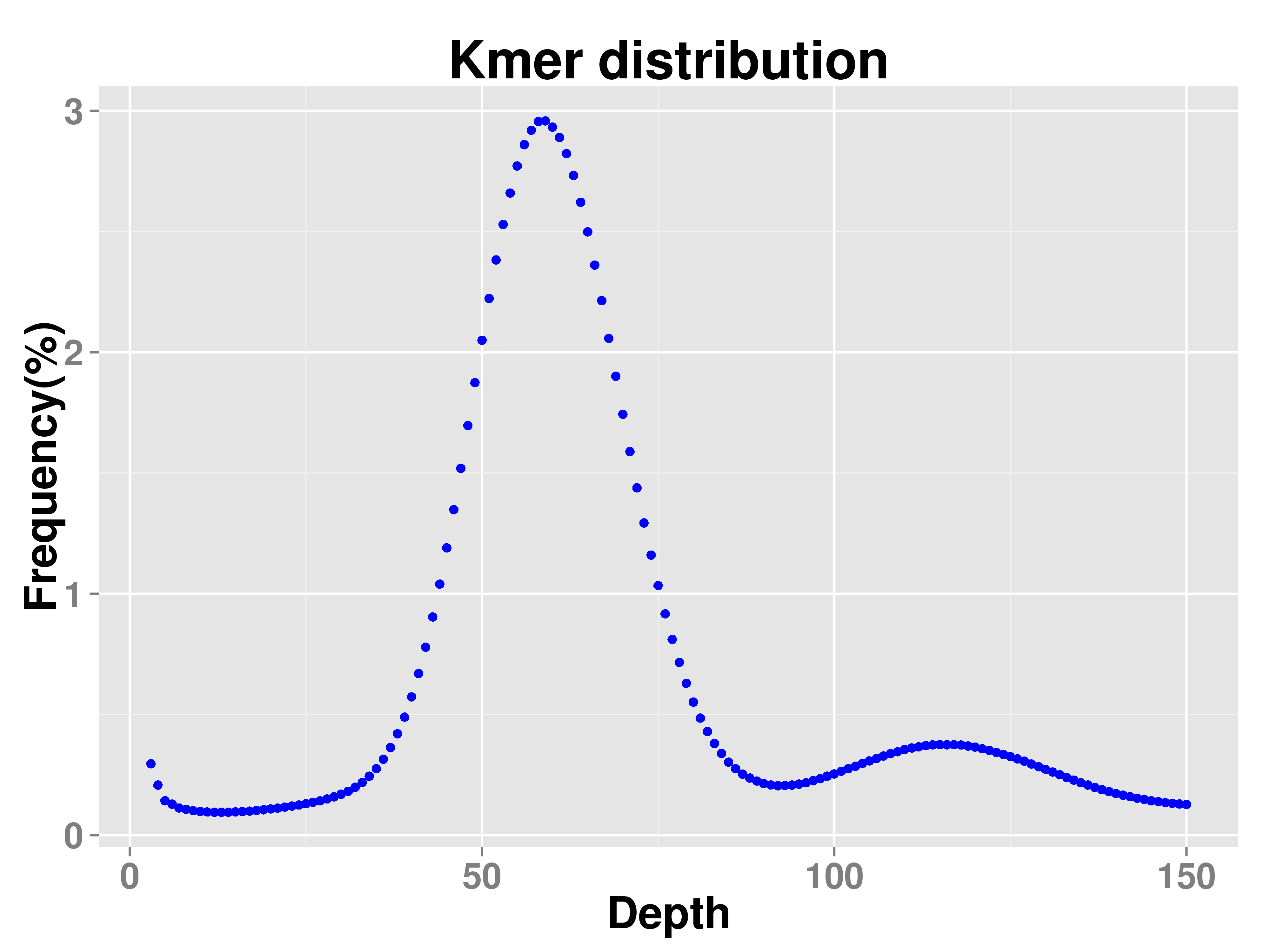


**Figure S4.** K-mer frequency distribution curve (k-mer=19) of Illumina short reads of the *Fragaria nilgerrensis* genome. The horizontal axis represents the depth of coverage of the K-mer, and the vertical axis represents the frequency of the depth of the K-mer. The blue line in the figure represents the frequency value.


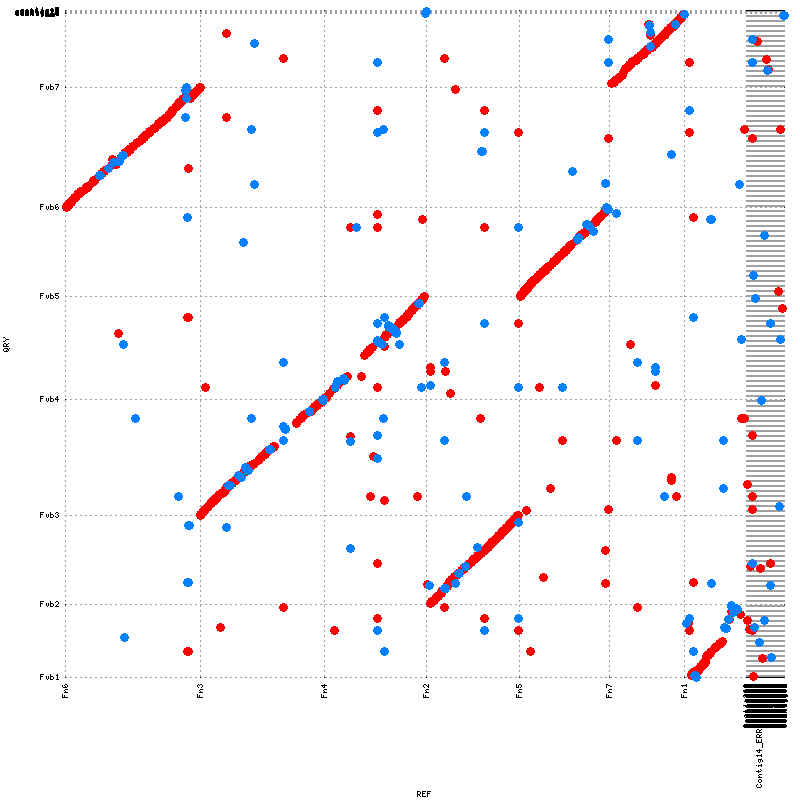


**Figure S5.** Gene collinearity between the *Fragaria veca* and *F. nilgerrensis* genomes. The x-axis corresponds to the *F. nilgerrensis* genome, and the y-axis corresponds to the *F. veca* genome


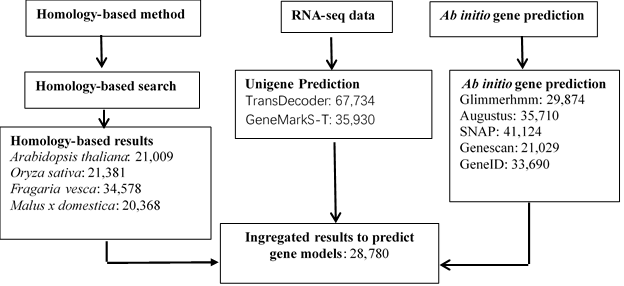


**Figure S6.** Schematic workflow for the gene annotation of the *Fragaria nilgerrensis*.


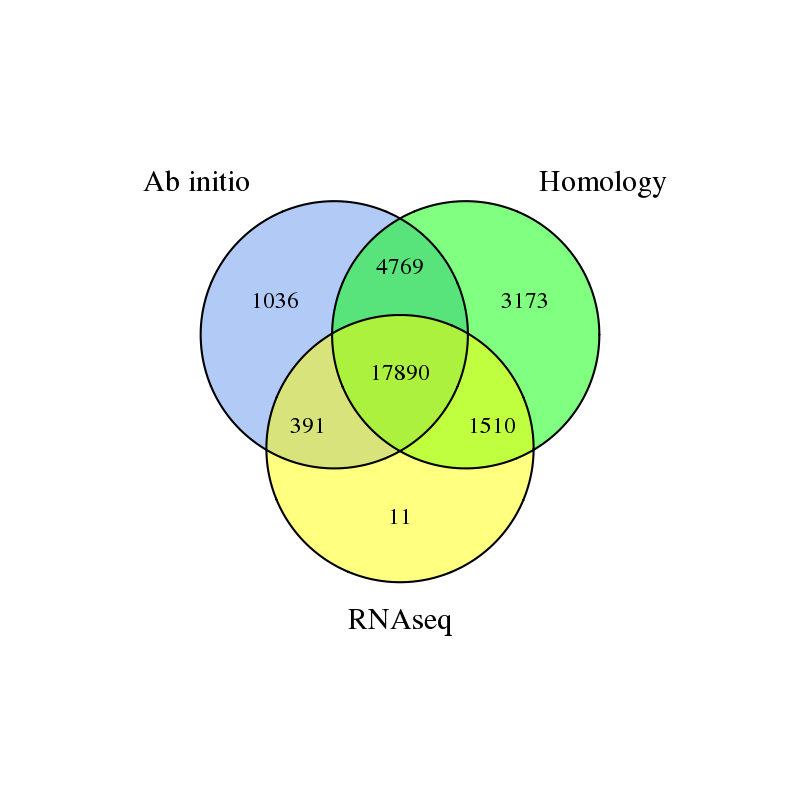


**Figure S7.** Venn diagram of gene annotation of the *Fragaria nilgerrensis* based *ab initio* gene prediction, homology-based method and RNA-seq.


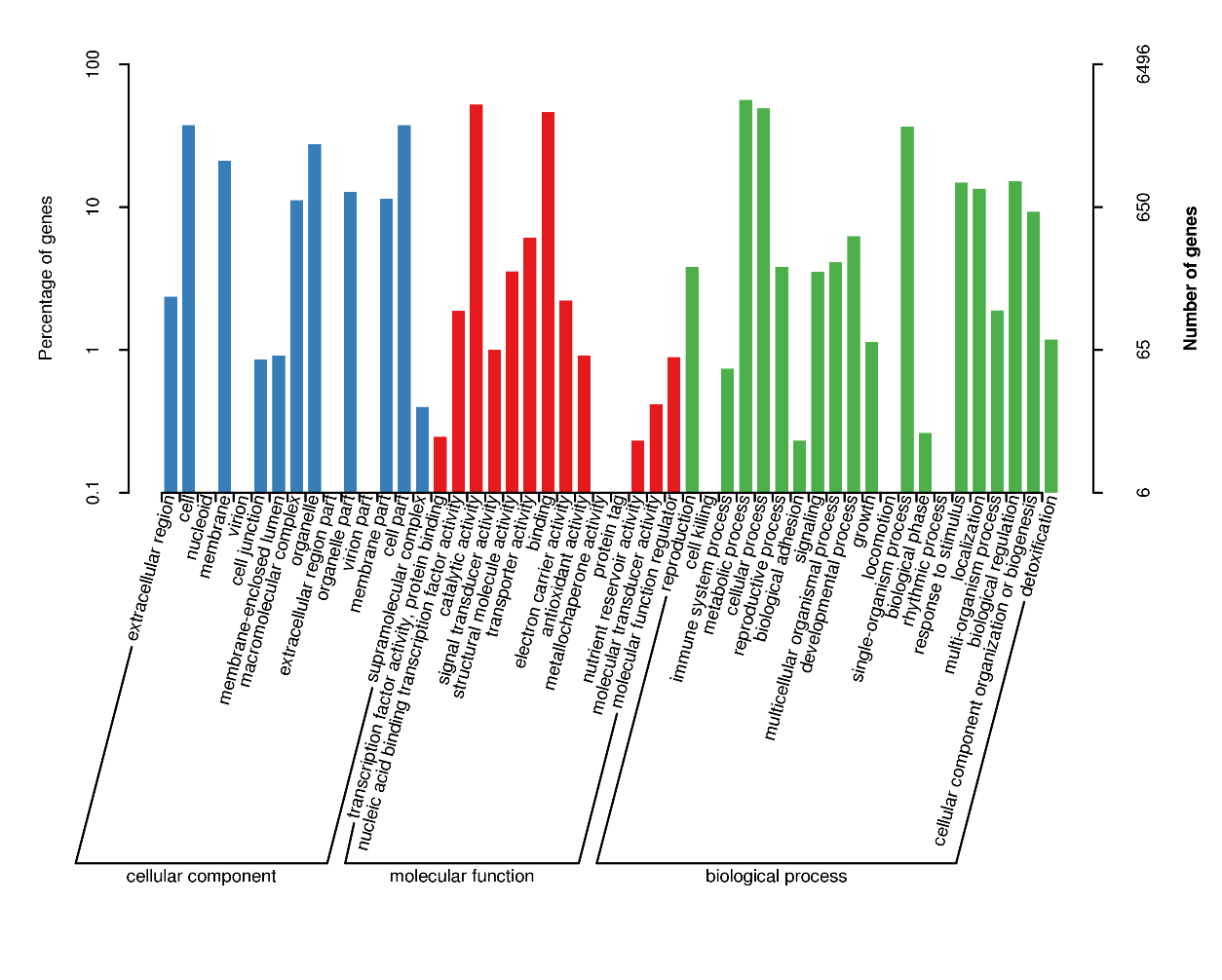


**Figure S8.** Gene ontology categories of the annotated genes. The y-axis represents the count of genes identified in this study. The genes were annotated in three main categories: cellular component, molecular function and biological process (x-axis).


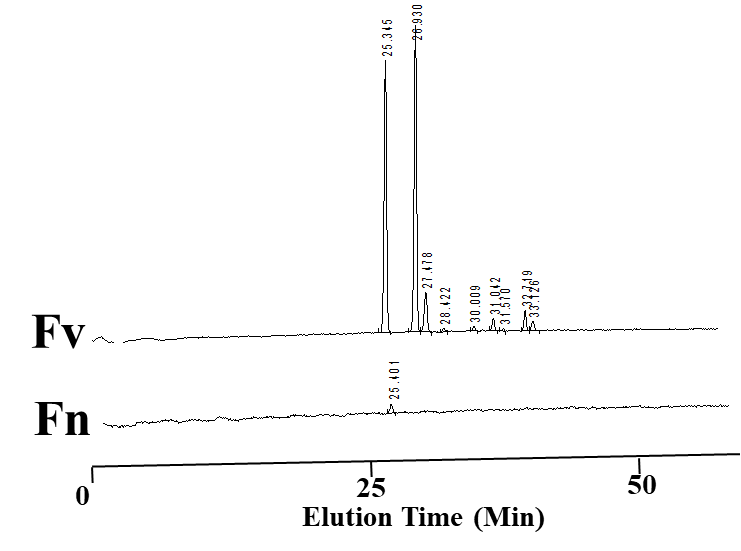


**Figure S9.** HPLC elution proﬁle of anthocyanins accumulated in the mature fruit of *Fragaria nilgerrensis* (Fn) and *F. vesca* (Fv). The absorbance was monitored at A520 nm.


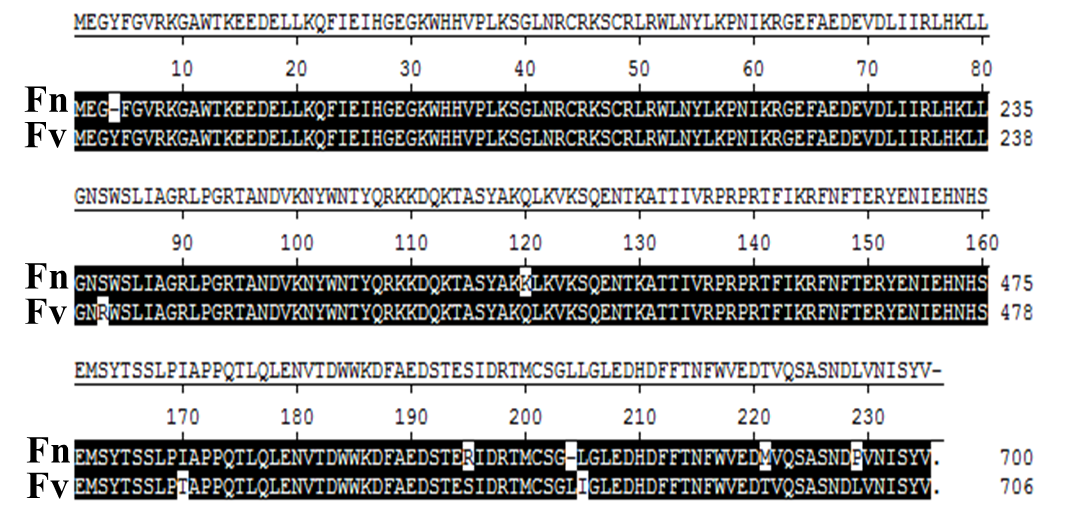


**Figure S10.** The alignment of MYB10 proteins from *Fragaria nilgerrensis* (Fn) and *F. vesca* (Fv) by DNASTAR software.


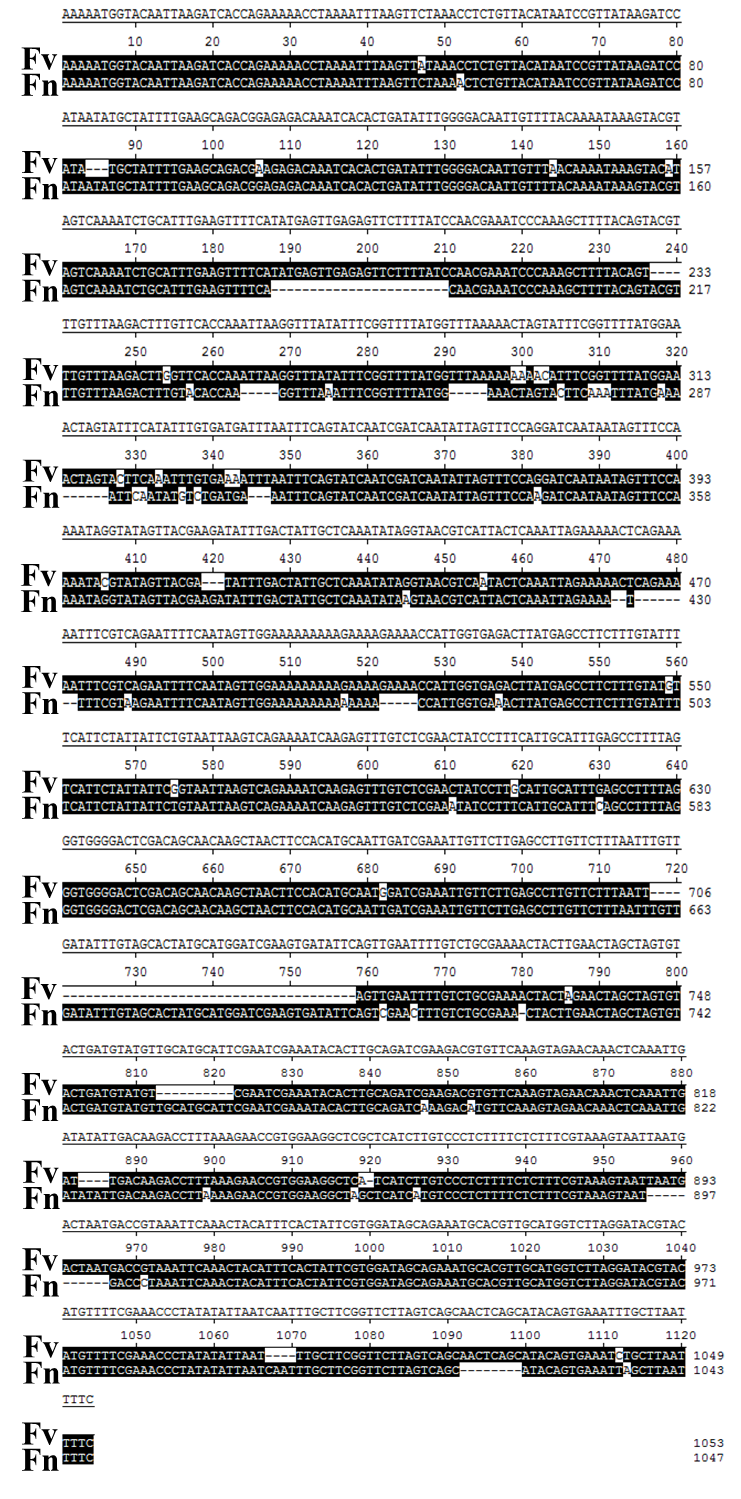


**Figure S11.** The alignment of MYB10 promoter sequences from *Fragaria nilgerrensis* (Fn) and *F.vesca* (Fv) by DNASTAR software.
