# Supplementary material for: The high‐quality genome of diploid strawberry (Fragaria nilgerrensis) provides new insights into anthocyanin accumulation
Source: Plant Biotechnol J. 2020 Feb 15;18(9):1908–24. doi: 10.1111/pbi.13351 (PMC7415782; doi:10.1111/pbi.13351)
Supplement: Supplementary file 2 — Table S1 Genome completeness evaluation of Fragaria nilgerrensis based on Illumina sequencing reads. Table S2 Genome completeness assessment of Fragaria nilgerrensis genome by BUSCO. Table S3 Completeness analysis of Fragaria nilgerrensis genome based on CEG database. Table S4 Gene prediction of Fragaria nilgerrensis based on Ab initio, Homology‐based and RNA‐seq method. Table S5 Gene functional annotation of Fragaria nilgerrensis based on different databases. Table S6 Noncoding RNA prediction of Fragaria nilgerrensis. Table S7 The number and percentage of SNPs types in Fragaria nilgerrensis compared with F. vesca. Table S8 The numbers of SNPs in different chromosome in Fragaria nilgerrensis compared with F. vesca. Table S9 The numbers of Indels in different chromosome in Fragaria nilgerrensis compared with F. vesca. Table S10 Primers used in this study. Appendix S1 The sequences of 373 orthologs in 14 species used for analysing the phylogenetic relationships. [file PBI-18-1908-s003.docx]

**Table S1.** Genome completeness evaluation based on Illumina sequencing reads.

| Library | Total reads | Mapped reads  to assembly | Mapped reads ratio (%) | Properly  mapped reads to assembly | Properly  mapped reads to assembly ratio (%) |
| --- | --- | --- | --- | --- | --- |
| 270 | 130,975,124 | 128,512,791 | 98.12 | 127,150,650 | 97.08 |

**Table S2.** Genome completeness assessment of *Fragaria* *nilgerrensis* genome by BUSCO.

| Categories | Number | Percent (%) |
| --- | --- | --- |
| Complete BUSCOs | 1292 | 89.72% |
| Complete and single-copy BUSCOs | 1216 | 84.44% |
| Complete and duplicated BUSCOs | 76 | 5.28% |
| Fragmented BUSCOs | 32 | 2.22% |
| Missing BUSCOs | 116 | 8.06% |
| Total BUSCO groups searched | 1440 | 100.00% |

**Table S3.** Completeness analysis of *Fragaria* *nilgerrensis* genome based on CEG database.

| Number^a^ (458 of CEGs) | Percent^a^ (%) | Number^b^ (248 of CEGs) | Percent^b^ (%) |
| --- | --- | --- | --- |
| 450 | 98.25% | 240 | 96.77% |

Note：**a**. Number and percentage of 458 core eukaryotic genes (CEGs) included in the genome assembly. **b**. Number and percentage of 248 highly conserved CEGs included in the genome assembly.

**Table S4.** Gene prediction of *Fragaria nilgerrensis* based on *Ab initio,* Homology-based and RNA-seq method.

| Method | Software | Species | Gene number |
| --- | --- | --- | --- |
| *Ab initio* | Genscan |  | 21,029 |
|  | Augustus |  | 35,710 |
|  | GlimmerHMM |  | 29,874 |
|  | GeneID |  | 33,690 |
|  | SNAP |  | 41,124 |
| Homology-based method | GeMoMa | *A. thaliana* | 21,009 |
|  |  | *O.* sativa | 21,381 |
|  |  | *F.* vesca | 34,578 |
|  |  | *M. domestica* | 20,368 |
| RNA-seq | PASA |  | 34,426 |
|  | TransDecoder |  | 67,734 |
|  | GeneMarkS-T |  | 35,930 |
| Integration | EVM |  | 28,780 |

**Table S5.** Gene functional annotation of *Fragaria nilgerrensis* based on different database.

| Database | Annotated number | Percentage (%) |
| --- | --- | --- |
| GO | 11,799 | 43.01% |
| KEGG | 8,346 | 30.42% |
| KOG | 13,441 | 48.99% |
| TrEMBL | 24,455 | 89.13% |
| NR | 25,234 | 91.97% |
| All | 26,343 | 96.01% |

**Table S6.** Noncoding RNA prediction of *Fragaria nilgerrensis.*

| Type | Number | Family |
| --- | --- | --- |
| rRNA | 302 | 4 |
| tRNA | 495 | 22 |
| miRNA | 58 | 20 |
| snRNA | 81 | 8 |
| snoRNA | 369 | 2 |

**Table S7.** The number and percentage of SNPs types in *Fragaria nilgerrensis* compared with

*F. vesca*.

| Types | Number/Percentage |
| --- | --- |
| Total SNPs | 4,561,825 |
| GT | 220,120/4.83% |
| GA | 692,359/15.18% |
| GC | 159,446/3.50% |
| TG | 223,014/4.89% |
| TC | 688,690/15.10% |
| TA | 298,638/6.55% |
| AC | 222,783/4.88% |
| AG | 687,650/15.07% |
| AT | 299,793/6.57% |
| CA | 218,791/4.80% |
| CG  CT | 158,533/3.48%  692,008/15.17 |

**Table S8.** The numbers of SNPs in different chromosome in *Fragaria nilgerrensis* compared with *F. vesca*.

| Chr 1 | Chr 2 | Chr 3 | Chr 4 | Chr 5 | Chr 6 | Chr 7 |
| --- | --- | --- | --- | --- | --- | --- |
| 435,611 | 636,565 | 790,604 | 692,220 | 643,030 | 865,301 | 493,938 |

**Table S9.** The numbers of Indels in different chromosome in *Fragaria nilgerrensis* compared with *F. vesca*.

| Chr 1 | Chr 2 | Chr 3 | Chr 4 | Chr 5 | Chr 6 | Chr 7 |
| --- | --- | --- | --- | --- | --- | --- |
| 81,083 | 122,272 | 140,678 | 122,123 | 121,265 | 162,046 | 95,681 |

**Table S10.** Primers used in this study.

| Primer Name | Primer Sequence(5’-3’) |
| --- | --- |
| **qRT-PCR** | |
| PAL1-qPCR-F | ATGGAGACCGTAACCCAGAAT |
| PAL1-qPCR-R | TGGCAGACTCATCCAGTTCA |
| PAL2-qPCR-F | ACTCCTCAATGGTAACATCACC |
| PAL2-qPCR-R | ATCACCCTCAATTCCGGCCAAC |
| CHI-qPCR-F | AGGAGGAAGAAGCCTTGGAG |
| CHI-qPCR-R | GAGACACCCCTCTTGTTCCA |
| DFR-qPCR-F | CTGGAGCGATGTCGAATTTT |
| DFR-qPCR-R | AGAGGCGAAAGTCCGGTAAT |
| TT19-qPCR-F | CTTGTGATCCTTCCCAGCAT |
| TT19-qPCR-R | CATGCAGGCCTATTGGAAAT |
| F3H-qPCR-F | AGGGTGGCTTCATCGTTTC |
| F3H-qPCR-R | GGCTGGGGGCATTTCGGGT |
| MYB10-qPCR-F | TAAGACCTCGACCACGAACC |
| MYB10-qPCR-R | GCTTGCCGATTGTACCGTAT |
| CHS-qPCR-F | GACATACCTGGTGCCGACTT |
| CHS-qPCR-F | AGTGCCTGTCCGACCAATAC |
| TTG1-qPCR-F | CTACCCTCTCTACGCCATGG |
| TTG1-qPCR-R | AAGGAGAGAATGTCGACCCG |
| C4H1-qPCR-F | GAAACTTAAGGGTTTGAATGGA |
| C4H1-qPCR-R | AGTTGTGACCTGTGTGCTTGC |
| 4CL3-qPCR-F | AATCCTATGGTGGCAGACTA |
| 4CL3qPCR-R | ACTCCAACTGGCCTTGTTTT |
| bHLH33-qPCR-F | GTTCCAAGGGATGTGAGCAC |
| bHLH33-qPCR-R | AGAATGAGCCTTTCCCACGA |
| GL3-qPCR-F | ATGGGTTTGCAGAGGAGTGA |
| GL3-qPCR-R | GTTCTTCCTGGCAACCCTTG |
| 4CL2-qPCR-F | GAACATCGCCAAGAGCCCCG |
| 4CL2-qPCR-R | TCTGGGAAGGGATTCGTTG |
| C4H2-qPCR-F | ATGGCTCATCTAATCACCAA |
| C4H2-qPCR-R | TTTCGAGCCAAGTTTGAGG |
| 4CL1-qPCR-F | CAGCCAGGAAAGTCGCCTCC |
| 4CL1-qPCR-R | TGCACATCAACTTGACCTCA |
| UFGT-qPCR-F | GCCTGGATCCGTTGTGTACT |
| UFGT-qPCR-R | CAAGATCAATGGCTGCTGAA |
| ANS-qPCR-F | CCTCAAACACCTTCCGACT |
| ANS-qPCR-R | CCTCCCTTCTTCTAATCCC |
| 26S-qPCR-F | TAACCGCATCAGGTCTCCAA |
| 26S-qPCR-R | CTCGAGCAGTTCTCCGACAG |
| **Cloning- proFnMYB10/ proFvMYB10-LUC** | |
| proFvMYB10-LUC-F | TTGATATCGAATTCCTGCAGcccgggAAAAATGGTACAATTAAGAT |
| proFvMYB10-LUC-R | GCGGCCGCTCTAGAACTAGTggatccGAAAATTAAGCAGATTTCAC |
| proFnMYB10-LUC-F | TTGATATCGAATTCCTGCAGcccgggAAAAATGGTACAATTAAGAT |
| proFnMYB10-LUC-R | GCGGCCGCTCTAGAACTAGTggatccGAAAATTAAGCTAATTTCACTG |
